# Programmable ferroelectric bionic vision hardware with selective attention for high-precision image classification

**DOI:** 10.1038/s41467-022-34565-2

**Published:** 2022-11-17

**Authors:** Rengjian Yu, Lihua He, Changsong Gao, Xianghong Zhang, Enlong Li, Tailiang Guo, Wenwu Li, Huipeng Chen

**Affiliations:** 1 Institute of Optoelectronic Display, National & Local United Engineering Lab of Flat Panel Display Technology, Fuzhou University, Fuzhou, 350002 China; 2 Fujian Science & Technology Innovation Laboratory for Optoelectronic Information of China, Fuzhou, 350100 China; 3 Shanghai Frontiers Science Research Base of Intelligent Optoelectronics and Perception, Institute of Optoelectronics, Department of Materials Science, Fudan University, Shanghai, 200433 China; 4 National Key Laboratory of Integrated Circuit Chips and Systems, Zhangjiang Fudan International Innovation Center, Fudan University, Shanghai, 200433 China

**Keywords:** Ferroelectrics and multiferroics, Semiconductors, Photonic devices

## Abstract

Selective attention is an efficient processing strategy to allocate computational resources for pivotal optical information. However, the hardware implementation of selective visual attention in conventional intelligent system is usually bulky and complex along with high computational cost. Here, programmable ferroelectric bionic vision hardware to emulate the selective attention is proposed. The tunneling effect of photogenerated carriers are controlled by dynamic variation of energy barrier, enabling the modulation of memory strength from 9.1% to 47.1% without peripheral storage unit. The molecular polarization of ferroelectric P(VDF-TrFE) layer enables a single device not only multiple nonvolatile states but also the implementation of selective attention. With these ferroelectric devices are arrayed together, UV light information can be selectively recorded and suppressed the with high current decibel level. Furthermore, the device with positive polarization exhibits high wavelength dependence in the image attention processing, and the fabricated ferroelectric sensory network exhibits high accuracy of 95.7% in the pattern classification for multi-wavelength images. This study can enrich the neuromorphic functions of bioinspired sensing devices and pave the way for profound implications of future bioinspired optoelectronics.

## Abstract (web summary)

Selective attention is an efficient processing strategy to allocate computational resources for pivotal optical information. Here, the authors propose a bionic vision hardware to emulate the behavior, showing a potential in image classification.

## Introduction

Vision is important sensory information of the brain, and more than 80% of the external information is received by the brain through the vision. To process such extensive information, the selective attention enables humans to manage the information effectively through processing the salient regions and suppressing the non-salient regions^1–3^. The selective attention allows to extracting most relevant optical information in crowed visual scenes containing multiple competing stimuli^4–6^. The operating mechanism is based on spatial locations and visual features, which are mediated by complex brain neural networks^7–9^. Correspondingly, if a neuron within the sensory field in visual cortex receives two competing stimuli, the attended stimulus has an advantage over the unattended stimulus^5^.

Inspired by biological system, previous efforts on hardware implementation of selective attention^1,6,10–14^, are based on CMOS and conventional transistors, whereas it takes up large footprint and high computational cost. Meanwhile, the sensory unit is separated from processing system, which leads to tremendous challenge to synchronously handle signals. Currently, integration of neuromorphic functions such as visual recognition^15–19^ and light adaptation^20–23^ into a compact optical sensing device have been presented, indicating that bioinspired device with less complexity has a bright prospect in the field of optoelectronics. Although these visual perception devices successfully perform some neuromorphic functions, they typically lack selective attention function of pivotal optical information, and the hardware implementation of optoelectronic device with selective attention is still a major challenge to facilitate the visual perception system. High-efficient and intelligent hardware implementation of selective visual attention can overcome the challenge of insufficient computing power during parallel processing of all the sensory data in a limited processing capacity system, which demands large dynamic range memory to interpret visual information^24^. P(VDF-TrFE) as a promising ferroelectric material with tunable remnant polarization can increase the memory states in the memory cell, obtained by charge accumulation and depletion by intermediate polarization states, so it can be very optimized material for artificial visual perception systems to process the sensory information.

In this study, a programmable ferroelectric bionic vision hardware with selective attention is fabricated using quantum dots (QDs) and ferroelectric material. Benefitting from the polarization of ferroelectric material, programmable photonic memory strength can be adjusted by modulation of energy barrier. By scaling up the photonic synapses to 5×5 arrays, the UV light information can be recorded and suppressed selectively by visual system with different polarization direction. The device under positive polarization has wavelength-dependent photo responsivity in the image processing, which allows high-precision image classification compared with unpolarized ferroelectric sensory network. This research proposed an effective way to mimic the human visual system, and has profound implications for future neuromorphic photoelectric electronics.

## Results

**Fig. 1:**
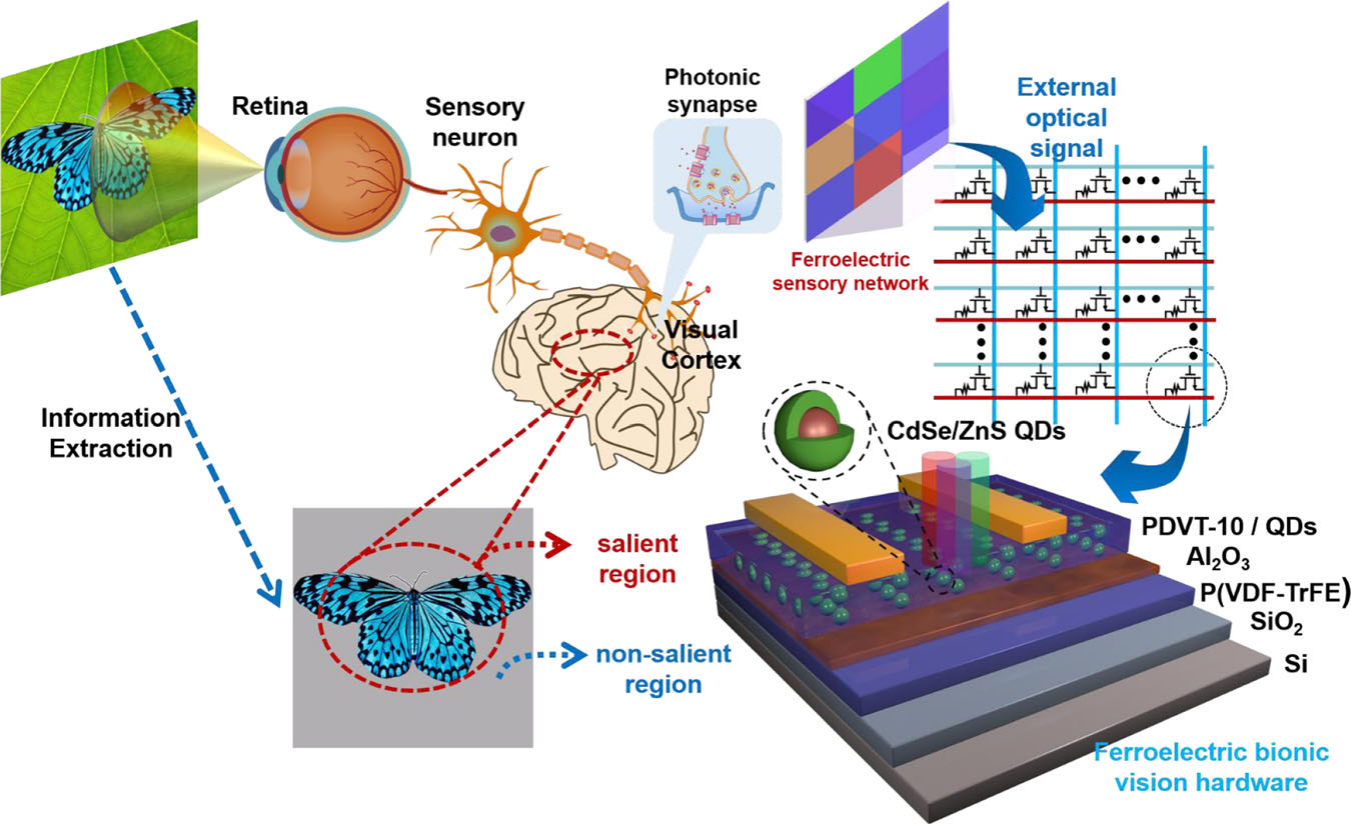
Schematic illustration of biological visual perception system and schematic configuration of individual artificial ferroelectric bionic vision hardware. The biological vision system converts visual information into electrical signals and processes the signals in the cortex. Information extraction is a significant function in the vision system, which is determined by synapses. So the ferroelectric bionic vision hardware (FeBVH) is proposed to play the roles of both sensor and visual processing.

The biological vision system as depicted in Fig. 1, which comprises retina, sensory neuron and visual cortex^25,26^, converts the visual information into electrical signals and processes the signals in the cortex. Information extraction is a significant function in vision system, which is determined by synapses. When external stimuli arrive at retina, the membrane potential of sensory neuron occurs due to the flux of Ca^2+^^27–30^. After that, neurotransmitters release from the presynaptic membrane and either strengthen or weaken the synaptic transition. The pivotal optical information in salient region has high synaptic weight in the visual cortex, while the synaptic weight of background information in non-salient region is low. Hence, the visual cortex can selectively screen and record the information that the brain concerns, and suppress the remaining information. To complete such complex function, the ferroelectric bionic vision hardware (FeBVH) is proposed to play the roles of both sensor and visual processing. The FeBVH converts external light into electrical output signals, while the pivotal and background optical information generate different photoresponse by light stimulations, which have a variety of intensities and wavelengths, so selective visual attention can be realized in FeBVH by constructing an array of ferroelectric transistors. The 3D schematic diagram of ferroelectric transistor shows bottom-gate top-contact structure. Particularly, PDVT-10 serves as the semiconductor layer, which is exposed to the light stimuli to investigate the photonic response characteristics of CdSe/ZnS quantum dots (QDs). Thus, the conductance increased by photogenerated holes generated from QDs, and the decay behavior of conductance by recombination of holes is similar to the Ca^2+^ dynamics in a synapse. In order to control the synaptic behavior to achieve the multi-state, the polarization characteristic of ferroelectric material is exploited in this device by using P(VDF-TrFE) as the ferroelectric layer. Organic ferroelectric material P(VDF-TrFE) has a higher degree of spontaneous polarization, excellent polarization stability, faster polarization reversal time, smaller leakage current and can be processed at low temperature, which is compatible with semiconductor technology^28,31–33^. Supplementary Fig. S1 shows the polarization and X-ray diffraction (XRD) image of P(VDF-TrFE), which illustrate the formation of β-phase P(VDF-TrFE). The ferroelectric materials’ coercive voltage (V_c_) can be served as the threshold voltage for the non-volatile memory and optoelectronics^24,34–36^. Consequently, the functions of biological synapses can be emulated in the device due to this similarity behavior between photonic synapse device and biological synapse.

**Fig. 2:**
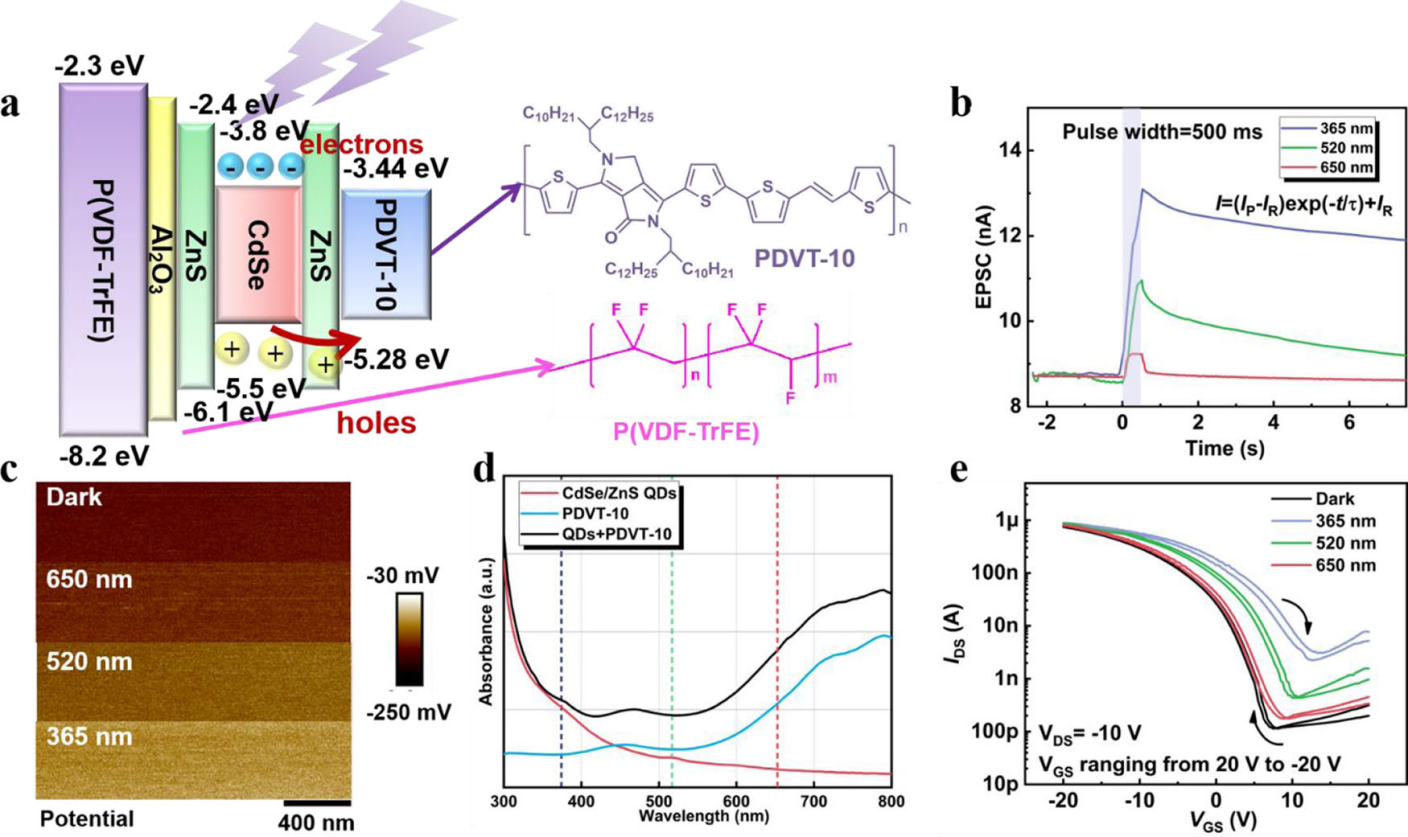
Selective attention of wavelengths. **a** The Holes generation process during the synaptic behaviors and the chemical structure of P(VDF-TrFE) and PDVT-10. **b** The excited postsynaptic current triggered by light pulses (1 μW/cm^2^, 0.5 s) with wavelengths of 365 nm, 520 nm and 650 nm. **c** Surface potential of PDVT-10/QDs composite film in the absence and presence of light illumination. **d** The absorption spectrum of CdSe/ZnS QDs, PDVT−10 and PDVT-10/QDs composite film. **e** The transfer curves of synaptic transistor illuminated by 365 nm, 520 nm and 650 nm (*V*_DS_ = −10 V, light intensity = 1 μW/cm^2^).

Figure 2a shows the process in the synaptic behaviors and the chemical structure of P(VDF-TrFE) and PDVT-10^31,37^. The values of energy levels are obtained from the literatures^37–40^. The QDs generate holes and electrons by light stimulation, and these holes can easily tunnel through low energy band between the QDs and semiconductor, which leads to the increase of channel conductance. However, electrons are hardly to trunnel through higher barrier between PDVT-10 and CdSe. At this time, it is difficult for electrons to recombine with holes, so the nonvolatile characteristic occurs. Supplementary Fig. S2 shows the morphological characterizations of films, indicating the evenly distribution of QDs in the PDVT-10 film. When the stimuli are removed, these photogenerated carriers recombine and the conductance decays slowly. Figure 2b demonstrates this phenomenon, and the excited postsynaptic current (EPSC) triggered by 365 nm increases rapidly and can reach up to 13 nA, which is much higher than the current triggered by 650 nm. What’s more, EPSCs with variable wavelengths of light perform the synaptic behaviors form short-term plasticity (STP) to long-term plasticity (LTP). In order to quantitatively the decay rate of the synaptic weight after applying light pulses with different wavelengths, an exponential equation is used to fit the current^41,42^

1 $$I=({I}_{{{{{{\rm{P}}}}}}}-{I}_{{{{{{\rm{R}}}}}}})\exp \left(-\frac{t}{\tau }\right)+{I}_{{{{{{\rm{R}}}}}}}$$

where *I*_P_ and *I*_R_ are the peak and retention current after the light pulse, and *τ* is the decay constant. When applying a 365 nm light pulse, the decay constant is determined to 29.9 s, which is much higher than the case of 600 nm (Supplementary Fig. S3). To further verify the illumination effect with different wavelengths, the surface potential of PDVT-10/QDs layer is measured by using Kelvin probe force microscopy (KPFM), as displayed in Fig. 2c. The surface potential remains almost consistent under 650 nm or 520 nm illumination, while it has obvious increase when the wavelength of light is 365 nm, indicating that the UV illumination can enhance the generation of carriers significantly. Meanwhile, according to the absorption intensity as presented in Fig. 2d, the different EPSC trends are primarily ascribed to the different absorption intensities for different light wavelengths. It exhibits high absorption intensity of QDs in the UV region. The double sweeping transfer curves with three wavelengths show a positive drift as the wavelength decreases, which further verifies the synaptic dynamics of FeBVH under different wavelength conditions. The detailed sweeping transfer curves are shown in Supplementary Fig. S4, almost no hysteresis is observed in the double sweeping curves with gate voltage ranging from −10 V to 10 V, showing that low gate voltage will not cause the polarization of the ferroelectric layer. Conversely, as gate voltage exceeds coercive voltage (*V*_c_) in the ferroelectric material, the ferroelectric material occurs polarization and the hysteresis is generated. Meanwhile, the double sweeping curves of ferroelectric device without Al_2_O_3_ layer show a counterclockwise hysteresis loop, which can be explained by the trapping effect can be reduced by insertion of Al_2_O_3_ layer.

**Fig. 3:**
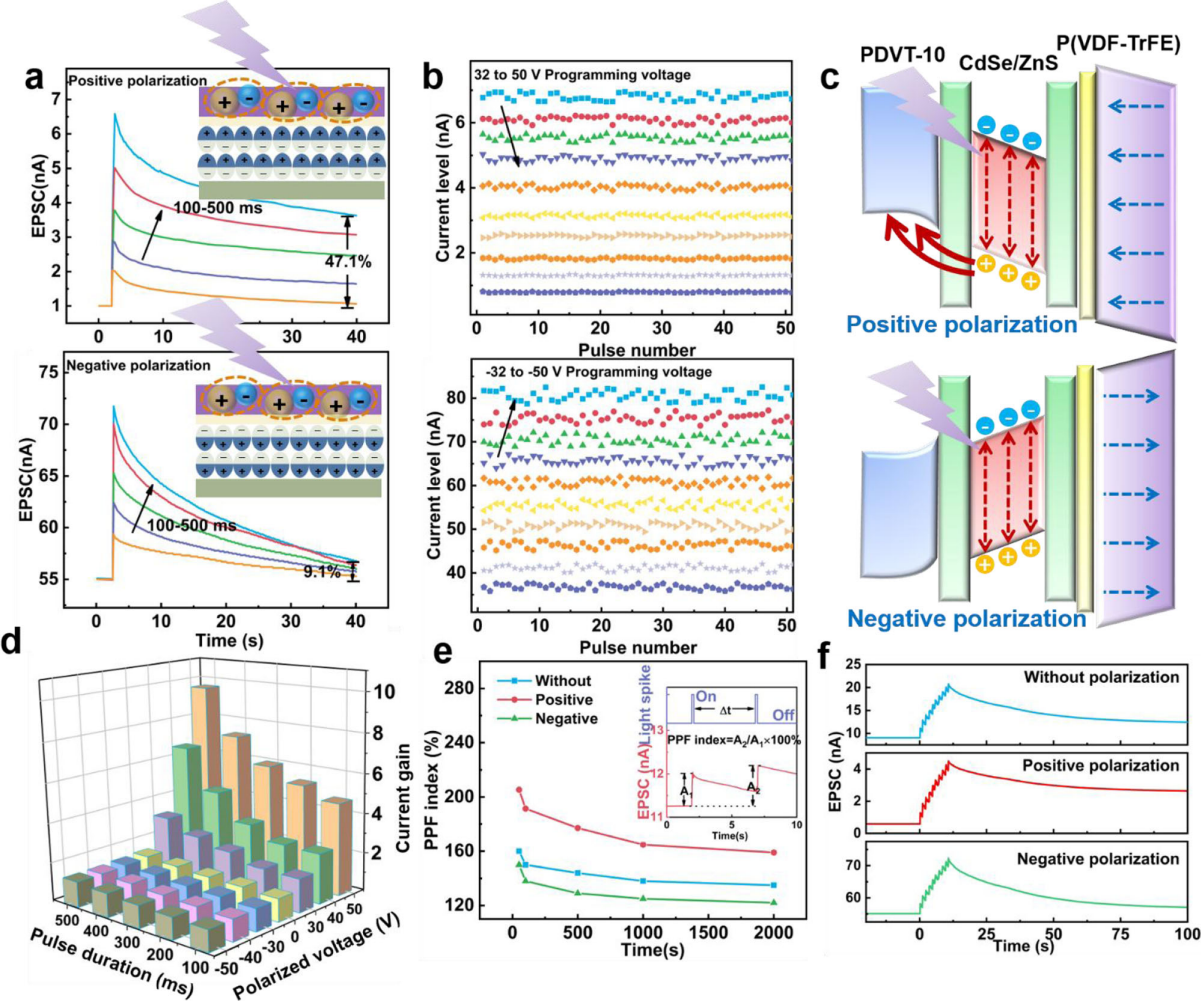
Selective attention under polarization. **a** The exciting postsynaptic current triggered by variant pulse durations (100, 200, 300, 400, and 500 ms) after 40 V polarization and –40 V polarization, where the ferroelectric materials’ coercive field can be exploited as photoelectric performance under UV light. **b** The retention current in the 10 states of EPSCs after positive polarization and negative polarization, where the device is polarized 50 times. **c** The transferring process of photogenerated carriers in the device after positive polarization and negative polarization. The excited postsynaptic current triggered by 100 ms light pulse in **d** Current gain as function of pulse width and polarized voltage. **e** EPSC triggered by a pair of light pulses and the paired pulse facilitation index as the function of pulse interval. **f** The current gain to 10 consecutive light pulses (1 µWcm^−2^, 100 ms) with and without the polarization.

As presented in Fig. 3a, by varying the gate voltage, the device can switch the polarization and the ferroelectric materials’ coercive field can be exploited as photoelectric performance (volatile and non-volatile) modulation under UV light. Here, as gate bias exceeds coercive positive voltage, the device is positively polarization and produces non-volatile photogenerated currents, while the device is negatively polarization and produces volatile photogenerated currents. A series of UV light pulses (1 μW/cm^2^) with different durations are applied to the device, which presents the current response with variable durations under positive (40 V polarization) and negative polarization (−40 V polarization), respectively. The memory factor (*η*_M_) can reflect the memory strength after light stimuli, which is defined as^43^

2 $${\eta }_{M}=\frac{{I}_{{{{{{\rm{R}}}}}}}-{I}_{0}}{{I}_{{{{{{\rm{P}}}}}}}-{I}_{0}} \times 100\%$$

where *I*_0_ is the original current. After positive polarization, the memory factor increases from 34.5% to 47.1%, which exhibits obvious LTP behavior, while current exhibits STP behavior (*η*_M_ = 9.1%) after negative polarization. By varying the duration of the optical pulse, the EPSC of FeBVH can be modulated from 2.5 nA to 6.5 nA. Moreover, EPSCs are triggered by the same light pulse by programming different degrees of polarized voltages. As shown in Fig. 3b, the 10 states of retention currents in 50 cycles of measurement are measured by applying 10 different positive bias pulses (32 to 50 V) to gate electrode. As the amplitude of positive bias increases, the polarization state of ferroelectric layer changes gradually from initial state to positive state, which results in the decrease of current with the increase of the retention time. In contrast, the 10 states of retention currents in negative polarization increase and the decay constant (Supplementary Fig. S5) decreases with the increase of polarization intensity. The retention currents in both positive and negative polarization maintain well-defined states in the programming cycles (Supplementary Figs. S6 and S7). Systematic measurements of current gain by varying the polarization intensity and duration of light pulses show that current gain is positively related to polarization intensity and duration of light pulses, as plotted in Fig. 3d. The mechanism of volatile and nonvolatile behavior and is attributed to the transfer of holes in the energy band illustrated in Fig. 3c, and the gradual change of multiple current states of transistor can be explained by partial polarization switching in the ferroelectric domains (Supplementary Fig. S8). The photogenerated holes can inject into the high occupied molecular orbital (HOMO) level of PDVT-10 spontaneously due to the energy level difference. The holes in the semiconductor layer are accumulated, leading to the increase of channel conductance. Then the holes dissipate and return to QDs gradually. As the positive voltage increases, the upward polarization domain gradually occurs, thereby increasing the current level. When the device is positive polarized, the photogenerated holes tunnel through the energy band more easily, while the positive polarization field impedes the recombination of photogenerated carriers, which leads to LTP in the device. Conversely, if the polarization is negative, the polarized electric field blocks the migration of the photogenerated holes from QDs to the semiconductor, and the holes in semiconductor dissipate rapidly, thereby manifesting the short-term relaxation characteristics, and polarization domains are aligned downward gradually with the increase of negative voltage.

Moreover, the synaptic properties of the devices under multiple optical pulses are tested. As a parameter quantifying the synaptic weight, paired pulse facilitation (PPF) represents the synaptic event stimulated by two consecutive presynaptic pulses^44,45^. It is a form of important short-term synaptic plasticity in processing of the synaptic signals, especially to decode the temporal information in the visual signals, which can be defined as^46^

3 $${{{{{\rm{PPF}}}}}}=\frac{{A}_{2}}{{A}_{1}}\times 100\%$$

Where *A*_2_ and *A*_1_ are EPSC amplitudes of first pulse and second pulse, respectively. Figure 3e shows PPF index as the function of pulse interval (Δt) under positive and negative polarization, which indicates that PPF decreases with the increase of Δt. And the enhanced PPF and depressed PPF can be observed under positive and negative polarization, respectively. The variation of relaxation time of devices can also be realized by applying multiple identical optical pulses in this device. Additionally, Fig. 3f indicates that the EPSC responses to 10 consecutive light pulses (1 µWcm^−2^, 100 ms) with and without the polarization. And the current gains which are defined as the ratio between the peak amplitudes after the *n*th optical pulse stimulation (*A*_*n*_) and the initial current (*A*_*0*_) are summarized in Supplementary Fig. S9. It exhibits linear enhancement and the current gain can reach up to 6.7, which is much higher than the case of negative polarization. In summary, when the device is negatively polarized, it exhibits high initial conductance, low current gain and short retention time. In contrast, when the device is positively polarized, the initial conductance is smaller, but the current gain and the retention time are larger. Consequently, the synaptic weight and the response to the optical pulses can be modulated by controlling the polarization direction of the ferroelectric layer, so that the device can achieve different functions.

**Fig. 4:**
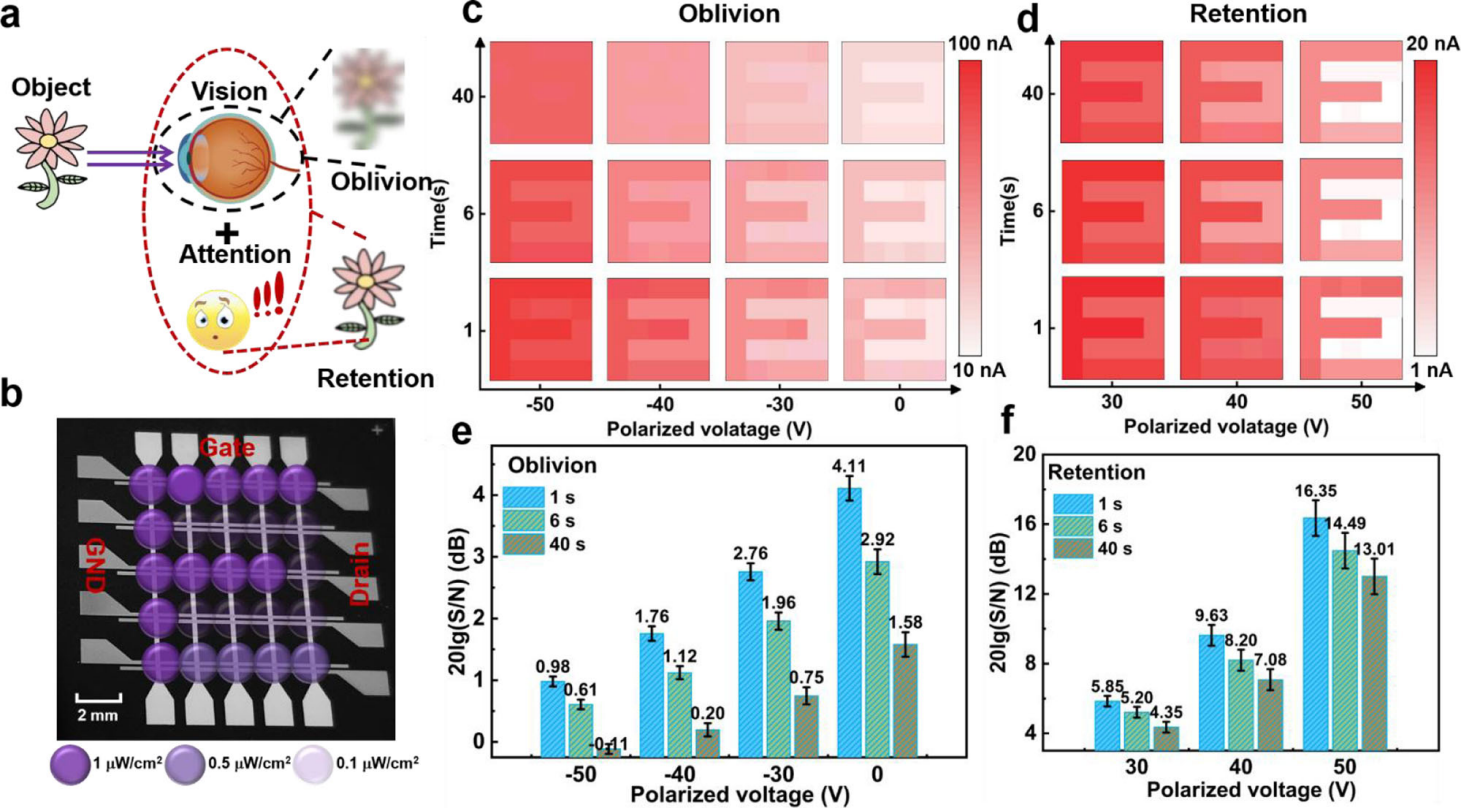
The artificial visual perception arrays to detect UV light information. **a** The schematic diagram of the object recognized by the visual system with the attention and without attention. **b** Optical microscopy image of the artificial visual perception including 5 × 5 pixel arrays. **c** The encoded images (output current) under negative polarization (−30, −40, −50 V) and without polarization (0 V). **d** The encoded images under positive polarization (30, 40, 50 V). The time-dependent signal to noise ratio of the perception arrays exposed under **e** negative polarization and **f** positive polarization.

In human biological system, only objects that are paid attention can be retained (“retention”) and most of visible unnoticed objects fade over (“oblivion”) in the memory, which is depicted in Fig. 4a. To testify the viability with selective attention in the developed device, the artificial visual perception including 5×5 arrays is fabricated as shown in Fig. 4b. The perception arrays are exposed to a UV signal of pattern “E” as shown in the optical microscopy image. In the perception arrays, the drain bias is −10 V and polarized voltage is applied to gate electrode. The EPSC of every pixel triggered by light pulses is measured under positive and negative polarization, as plotted in Fig. 4c, d, respectively. (Supplementary Figs. S10–12). By applying negative polarization, the signal cannot be clearly discriminated after 40 s, showing a lower contrast between the signal and background noise compared to the image without polarization. In contrast, the signal can be clearly discriminated after positive polarization, and the signal is easier to discriminate with the increase of polarized intensity. To evaluate the contrast, the signal-to-noise ratio (SNR) can be addressed for the perception arrays, which is defined as

4 $${{{{{\rm{SNR}}}}}}=20{{{{{\rm{lg}}}}}}\left(\frac{S}{N}\right)$$

where S and N are the currents of signal and noise. As shown in Fig. 4e, f, the time-dependent SNR decreases(SD error bar) with the increase of the time after light pulses. Compared to low SNR in the negative polarization, the retentional SNR (40 s) of signals can respectively reach 13.0 dB after 50 V positive polarization. These current decibel levels exhibit obvious contrast difference of up to 13.12 dB under positive and negative polarization. As a result, the perception arrays can provide the viability with selective attention of pivotal optical information in a complicated environment, which is determined by the polarized direction.

**Fig. 5:**
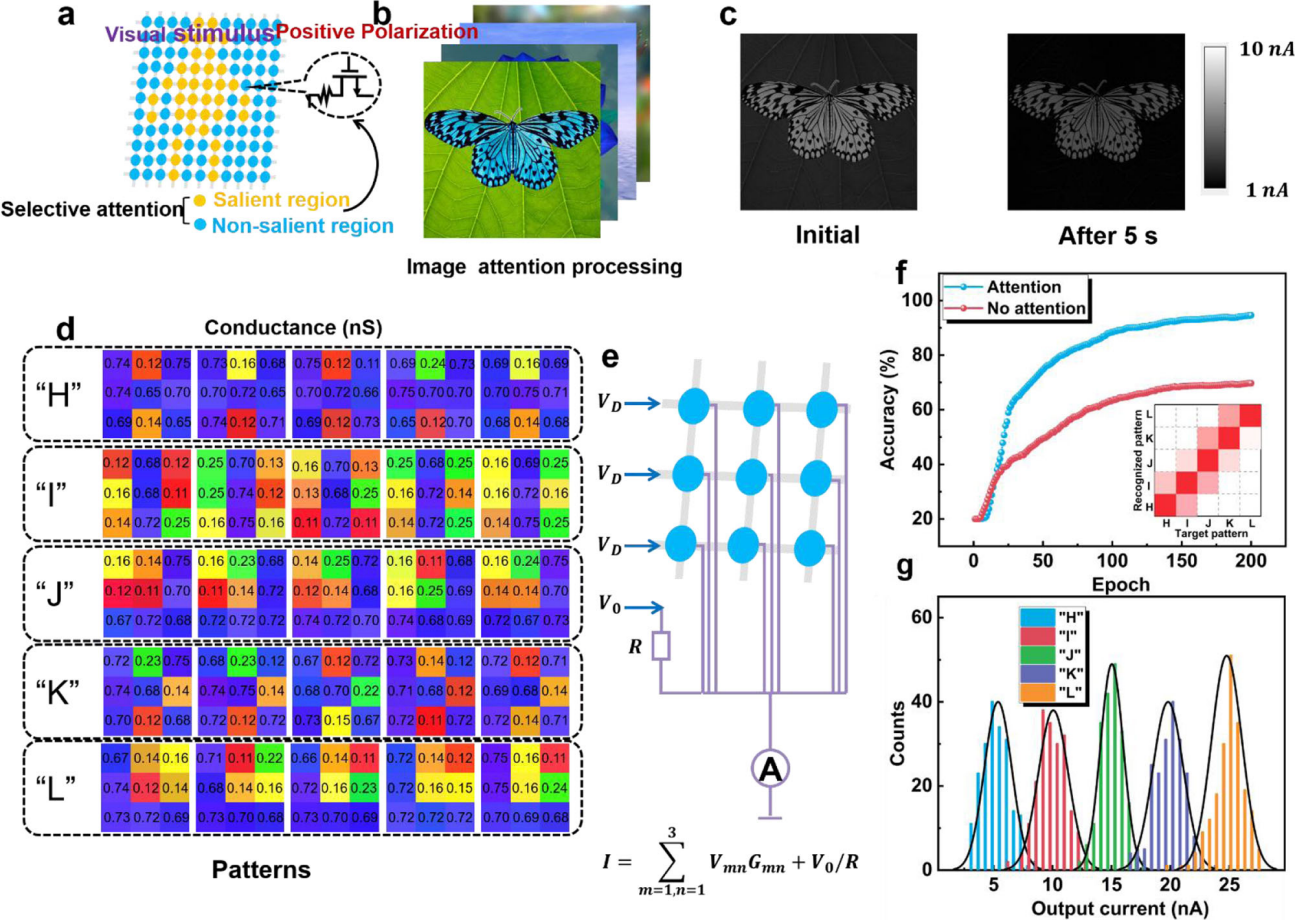
Image attention processing and pattern classification. **a** Schematic illustration of the structure of image processing array. **b** Processing images with short wavelengths and long wavelengths. **c** The realization of image attention processing with the increased of decay time. **d** The detected patterns of “H”, “I”, “J”, “K”, “L” letters with different wavelengths and encoding conductance of pattern with variant wavelengths. **e** Schematic diagram device array to realize the multiplication-accumulation operation. **f** Accuracy of multiplication-accumulation circuits with the increase of training epoch. **g** The distribution of the output accumulated current in the 5 input patterns.

With the selective attention effect of the device, the image attention processing is implemented with the artificial visual perception arrays, which is illustrated in Fig. 5a. Under positive polarization, the light modulation with different wavelengths determines the photoresponse, and therefore the short-wavelength signals can be extracted from a complicated environment. As a proof of concept, the photocurrent of each pixel is measured by a single device one by one to perform image attention processing, where light stimulation of per pixel is considered as superposition of three monochrome light (Supplementary Fig. S13). As shown in Fig. 5b, an image contains a blue “butterfly” and a green “leaf”. It is worth noting that short-wavelength signals obtain large response in our device. At the beginning, the “butterfly” is mainly highlighted and the feature of “leaf” is suppressed. After 5 s, the feature of“leaf” is completely absent and the “butterfly” is extracted.

Not limited to extraction of pivotal optical information, the image attention processing can be used to sensory network to classify specific images and achieve higher accuracy. The training image dataset is presented in Fig. 5d, which includes 5 different patterns of letters (“H”, “I”, “J”, “K”, “L”). The training images consist of variant wavelengths of signals (365 nm, 420 nm, 520 nm, 570 nm, 600 nm and 650 nm) where the short-wavelength signals (365 nm and 420 nm) serve as pivotal information. After positive polarization, the distribution of conductance in our device under different wavelengths demonstrates that the conductance under blue and UV light obtain remarkable difference than other lights. For the classification to these letters, as depicted in Fig. 5e, a simple neural network is built by multiply-accumulate circuits (MAC)^47,48^, where structure of circuit is shown in Supplementary Fig. S14, since the drain current is linear in the measured range (−20V to 20 V) of drain voltage under the light illumination (Supplementary Fig. S15). Every device serves as the input neuron, and the drain voltages are considered as the weights of neural network. According to Kirchhoff’s and Ohm’s laws, the output current can be described as

5 $${I}_{{out}}=\mathop{\sum }\limits_{m=1,n=1}^{3}{V}_{{mn}}{G}_{{mn}}+{V}_{0}/R$$

where *V*_*mn*_ and *G*_*mn*_ are drain voltage and conductance in *n* column of *m* row, and *V*_*0*_/*R* is the bias current to accelerate the fitting of neural network. In the training epoch, the input pattern is encoded to conductance and incorporated into the neural network to update the drain voltage, where the flow charts of training and testing process are demonstrated in Supplementary Fig. S16. The training process aims to train appropriate weight values to decrease the loss function (Supplementary Fig. S17). In the simulation, as shown in Fig. 5h, our device can reach 95.7% accuracy with the increase of training epoch, where the training result is provided in Supplementary Table S1. Compared with the accuracy (69.7%) of classification without selective attention summarized in Supplementary Fig. S18, the accuracy can be greatly improved. Figure 5g demonstrates the distribution of output current when different patterns are incorporated into the neural network, so the small overlap of distribution shows that the pattern can be determined precisely according to the output current. Due to the integration of sensory and computing function, hardware overhead of the neural network is lower than other works^49–56^. (Supplementary Fig. S19).

Finally, the superiorities of FeBVH includes: (i) Tunable photoresponse. The ferroelectric layer endows the device with programmable nonvolatile synaptic states, which enables hardware implementation of optoelectronic device with selective attention. (ii) Less hardware overhead. Both signed weight representation and light detection are enabled in a single device, so in-sensor neuromorphic function can be implemented in the ferroelectric sensory network, reducing the hardware overhead in the neural network. (iii) High accuracy. The FeBVH-based sensory network with selective attention can obtain a high-precision of 95.7% accuracy in the classification of letters, which is much higher than that of the network without selective attention (69.7%).

## Discussion

In Summary, we propose ferroelectric-based programmable bionic vision hardware to mimic the biological visual information processing in a single device. The ferroelectric layer endows the device tunable, nonvolatile and programmable synaptic states so that external visual information can be selectively recorded. As a result, the artificial visual perception arrays fabricated by the devices achieve the viability with selective attention of UV optical information determined by different polarization direction. The linear drain current and high wavelength dependence of the device enable the implement of the neural network for image recognition and classification, and the accuracy can increase greatly from 69.7% to 95.7% with selective attention. This work expands the neuromorphic functions of artificial visual perception system and offers a promising potential for future application in neuromorphic devices.

## Method

### Device fabrication

Copolymer poly(vinylidene fluoride-trifluoroethylene), P(VDF-TrFE) (70:30) purchased from Wuhan methyl technology co. LTD is dissolved in dimethyformamide(DMF) at a mass fraction of 6.15wt.%. The P(VDF-TrFE) film is deposited on cleaned Si substrate with a 100 nm oxidation layer at a spin coating speed of 2000 rpm for 60 s, and the film is annealed in the glove box at 80 °C for 0.5 h before annealing at 120 °C for 2 h. Then, Al_2_O_3_ layer with 10 nm is deposited by atomic layer deposition (ALD) to improve the roughness of interface. The semiconductor layer poly [2, 5-bis (alkyl) pyrrolo-[3, 4-c]pyrrole-1, 4 (2H, 5H)-dione-alt-5, 50-di (thiophen-2-yl)−2, 20-(E)−2- (2- (thiophen-2-yl)vinyl)thiophene] (PDVT-10, Mw = 183 kDa) and CdSe/ZnS quantum dots (QDs) are purchased from1-Material and Wuhan Jiayuan Quantum Dots Co., Ltd., respectively, and both are mixed at a weight ratio of 5:2, and the PDVT-10/QDs mixed solution is stirred for at least 12 h to ensure uniform dispersion. After that, the mixed solution is deposited on the Al_2_O_3_ by spin coating at 1000 rpm for 60 s. Finally, 50-nm Au electrode is deposited on the semiconductor layer by evaporation through a shadow mask.

### Equipment and characterization

All electrical properties of the device are measured by using a semiconductor parameter analyzer (Keithley B2912A) in an ambient atmosphere. The surface potential of film is examined by Kelvin probe force microscopy (KPFM, Bruke MultiMode 8) and UV–Vis absorption spectra of the film is measured by ultraviolet-visible near infrared spectrophotometer (Shimadzu UV-3600 Plus). The monochromatic light is generated by wavelength-adjustable xenon lamp source (Beijing NBET Technology Co., Ltd., Omno302) and the duration of light pulse is adjusted by a shutter. In the current measurements of visual perception arrays, the UV light generated by lamp source is transmitted through the optical fiber, and the convex lens converts the light to the parallel light. The device is illuminated though a hollowed mask with a “E” image. The polarization density of PVDF-TrFE thin film is measured using a pulse measurement unit (4225-PMU, KEITHLEY). The information extraction of image is simulated by Matlab software (Supplementary Fig. S12).

## Supplementary information

Supplementary Information

## Data Availability

The data that support the findings of this study are available from the corresponding author upon request.

## Code availability

The codes used for the simulations are available in [https://github.com/RengjianYu/NCOMMS-NCMS-22-29688].

## References

[1] Bartolozzi C, Indiveri G (2006). Selective attention implemented with dynamic synapses and integrate-and-fire neurons. Neurocomputing.

[2] Green CS, Bavelier D (2003). Action video game modifies visual selective attention. Nature.

[3] Linden MA, Crothers IR, Rauch RJ (2006). Exploring eye movement analysis as a measure of selective visual attention in brain injured individuals. Brain Inj..

[4] Moran J, Desimone R (1985). Selective attention gates visual processing in the extrastriate cortex. Science.

[5] Fries P, Reynolds JH, Rorie AE, Desimone R (2001). Modulation of oscillatory neuronal synchronization by selective visual attention. Science.

[6] Knudsen EI (2018). Neural circuits that mediate selective attention: A comparative perspective. Trends Neurosci..

[7] Moore T, Zirnsak M (2017). Neural mechanisms of selective visual attention. Annu. Rev. Psychol..

[8] Pagnotta MF, Pascucci D, Plomp G (2022). Selective attention involves a feature-specific sequential release from inhibitory gating. Neuroimage.

[9] You WK, Mysore SP (2020). Endogenous and exogenous control of visuospatial selective attention in freely behaving mice. Nat. Commun..

[10] Bartolozzi C, Indiveri G (2009). Selective attention in multi-chip address-event systems. Sensors.

[11] Carota L, Indiveri G, Dante V (2004). A software–hardware selective attention system. Neurocomputing.

[12] Giacomo I (2001). A neuromorphic VLSI device for implementing 2-D selective attention systems. IEEE T. Neural Netw..

[13] Cottini N, Gottardi M, Massari N, Passerone R (2013). A bio-inspired APS for selective visual attention. IEEE Sens. J..

[14] Roe DG (2021). Biologically plausible artificial synaptic array: Replicating Ebbinghaus’ memory curve with selective attention. Adv. Mater..

[15] Xie D (2018). Coplanar multigate MoS_2_ electric-double-layer transistors for neuromorphic visual recognition. ACS Appl. Mater. Interfaces.

[16] Seo S (2018). Artificial optic-neural synapse for colored and color-mixed pattern recognition. Nat. Commun..

[17] Huang X (2021). Dual-mode learning of ambipolar synaptic phototransistor based on 2D perovskite/organic heterojunction for flexible color recognizable visual system. Small.

[18] Li E (2021). High-density reconfigurable synaptic transistors targeting a minimalist neural network. ACS Appl. Mater. Interfaces.

[19] Fan T (2020). Analog sensing and computing systems with low power consumption for gesture recognition. Adv. Intell. Syst..

[20] Kwon SM (2019). Environment-adaptable artificial visual perception behaviors using a light-adjustable optoelectronic neuromorphic device array. Adv. Mater..

[21] Liao F (2022). Bioinspired in-sensor visual adaptation for accurate perception. Nat. Electron..

[22] Kumar M, Lim J, Kim S, Seo H (2020). Environment-adaptable photonic-electronic-coupled neuromorphic angular visual system. ACS Nano.

[23] Wu XM (2021). Artificial multisensory integration nervous system with haptic and iconic perception behaviors. Nano Energy.

[24] Wang H (2018). A ferroelectric/electrochemical modulated organic synapse for ultraflexible, artificial visual-perception system. Adv. Mater..

[25] Chen S, Lou Z, Chen D, Shen G (2018). An artificial flexible visual memory system based on an UV-motivated memristor. Adv. Mater..

[26] Duan N (2019). An electro-photo-sensitive synaptic transistor for edge neuromorphic visual systems. Nanoscale.

[27] Kim MK, Lee JS (2020). Synergistic improvement of long-term plasticity in photonic synapses using ferroelectric polarization in hafnia-based oxide-semiconductor transistors. Adv. Mater..

[28] Lee HR, Lee D, Oh JH (2021). A hippocampus-inspired dual-gated organic artificial synapse for simultaneous sensing of a neurotransmitter and light. Adv. Mater..

[29] Jiang J (2019). 2D electric-double-layer phototransistor for photoelectronic and spatiotemporal hybrid neuromorphic integration. Nanoscale.

[30] Yang F (2021). Vertical-organic-nanocrystal-arrays for crossbar memristors with tuning switching dynamics toward neuromorphic computing. SmartMat.

[31] He L (2021). Complementary of ferroelectric and floating gate structure for high performance organic nonvolatile memory. Adv. Electron. Mater..

[32] Li E (2019). Flexible ultra-short channel organic ferroelectric non-volatile memory transistors. J. Mater. Chem. C..

[33] Li E (2021). Nanoscale channel organic ferroelectric synaptic transistor array for high recognition accuracy neuromorphic computing. Nano Energy.

[34] Zhao Q (2017). Organic ferroelectric-based 1T1T random access memory cell employing a common dielectric layer overcoming the half-selection problem. Adv. Mater..

[35] Boyn S (2017). Learning through ferroelectric domain dynamics in solid-state synapses. Nat. Commun..

[36] Kim MK, Lee JS (2019). Ferroelectric analog synaptic transistors. Nano Lett..

[37] Lao J (2022). Ultralow-power machine vision with self-powered sensor reservoir. Adv. Sci.

[38] Reiss P, Protiere M, Li L (2009). Core/shell semiconductor nanocrystals. Small.

[39] Liu B (2022). Cadmium-doped zinc sulfide shell as a hole injection springboard for red, green, and blue quantum dot light-emitting diodes. Adv. Sci..

[40] Chen H (2012). Highly pi-extended copolymers with diketopyrrolopyrrole moieties for high-performance field-effect transistors. Adv. Mater..

[41] Yu R (2021). Bi-mode electrolyte-gated synaptic transistor via additional ion doping and its application to artificial nociceptors. Mater. Horiz..

[42] Gao J (2021). Intrinsic polarization coupling in 2D α-In_2_Se_3_ toward artificial synapse with multimode operations. SmartMat.

[43] Xie D (2022). Polarization-perceptual anisotropic two-dimensional ReS_2_ neuro-transistor with reconfigurable neuromorphic vision. Mater. Horiz..

[44] Guo F (2021). Multifunctional optoelectronic synapse based on ferroelectric van der Waals heterostructure for emulating the entire human visual system. Adv. Func. Mater..

[45] Ahmed T (2019). Optically stimulated artificial synapse based on layered black phosphorus. Small.

[46] Ercan E, Lin YC, Yang WC, Chen WC (2021). Self-assembled nanostructures of quantum dot/conjugated polymer hybrids for photonic synaptic transistors with ultralow energy consumption and zero-gate bias. Adv. Func. Mater..

[47] Migliato Marega G (2022). Low-power artificial neural network perceptron based on monolayer MoS_2_. ACS Nano.

[48] Cui B (2022). Ferroelectric photosensor network: An advanced hardware solution to real-time machine vision. Nat. Commun..

[49] Li, S. et al. Wafer-scale 2D hafnium diselenide based memristor crossbar array for energy-efficient neural network hardware. *Adv. Mater*. **34**, 2103376 (2021). 10.1002/adma.202103376 34510567

[50] Tang J (2019). Bridging biological and artificial neural networks with emerging neuromorphic devices: Fundamentals, progress, and challenges. Adv. Mater..

[51] Choi S (2018). Sige epitaxial memory for neuromorphic computing with reproducible high performance based on engineered dislocations. Nat. Mater..

[52] van de Burgt Y (2017). A non-volatile organic electrochemical device as a low-voltage artificial synapse for neuromorphic computing. Nat. Mater..

[53] Kim S (2017). Pattern recognition using carbon nanotube synaptic transistors with an adjustable weight update protocol. ACS Nano.

[54] Kim S, Yoon J, Kim HD, Choi SJ (2015). Carbon nanotube synaptic transistor network for pattern recognition. ACS Appl Mater. Interfaces.

[55] Liu, J. et al. Compensated ferrimagnet based artificial synapse and neuron for ultrafast neuromorphic computing. *Adv. Func. Mater*., **32**, 2107870 (2021).

[56] Yang C-S (2018). All-solid-state synaptic transistor with ultralow conductance for neuromorphic computing. Adv. Func. Mater..

