# Supplementary material for: Programmable ferroelectric bionic vision hardware with selective attention for high-precision image classification
Source: Nat Commun. 2022 Nov 17;13:7019. doi: 10.1038/s41467-022-34565-2 (PMC9669032; doi:10.1038/s41467-022-34565-2)
Supplement: Supplementary file 1 — Supplementary Information [file 41467_2022_34565_MOESM1_ESM.pdf]

**Supplementary Information**

# Programmable ferroelectric bionic vision hardware with selective attention for high-precision image classification

*Rengjian Yu[1,2], Lihua He[1], Changsong Gao[1], Xianghong Zhang[1], Enlong Li[3,4], Tailiang Guo[1,2], Wenwu Li[3,4]*, and Huipeng Chen[1,2]**

[1]Institute of Optoelectronic Display, National & Local United Engineering Lab of Flat Panel Display Technology, Fuzhou University, Fuzhou 350002, China

[2]Fujian Science & Technology Innovation Laboratory for Optoelectronic Information of China, Fuzhou 350100, China

[3]Shanghai Frontiers Science Research Base of Intelligent Optoelectronics and Perception, Institute of Optoelectronics, Department of Materials Science, Fudan University, Shanghai 200433, China.

[4]National Key Laboratory of Integrated Circuit Chips and Systems, Zhangjiang Fudan International Innovation Center, Fudan University, Shanghai 200433, China.

Email: liwenwu@fudan.edu.cn or hpchen@fzu.edu.cn

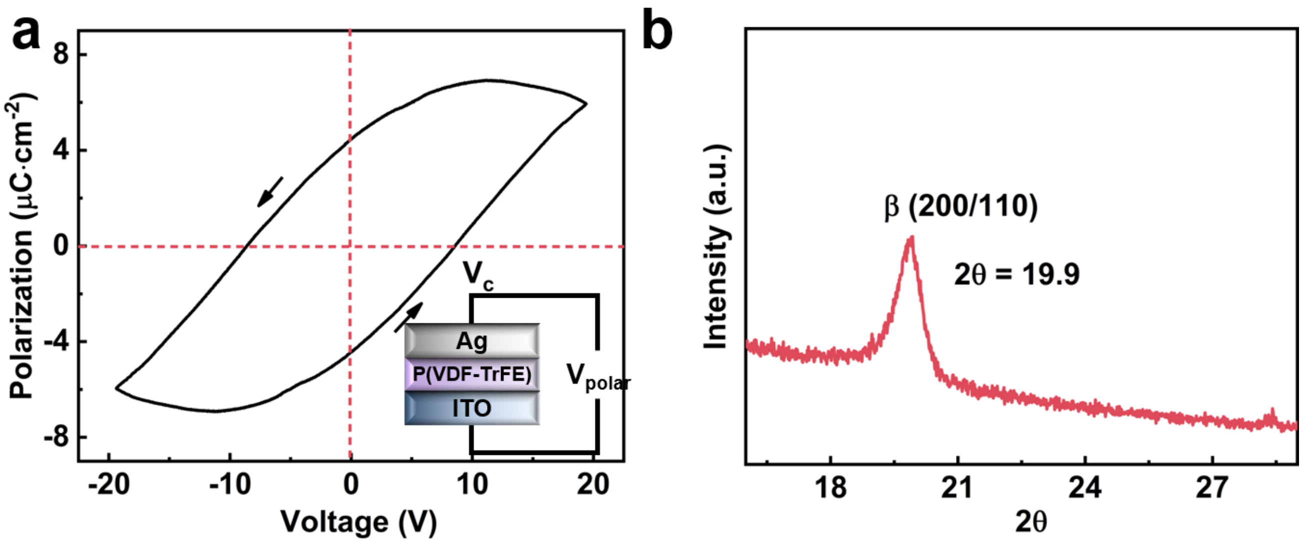


**Figure S1** a) Polarization−voltage (*P*−*V*) hysteresis curve and b) The XRD image of annealed PVDF-TrFE layer.

Fig. S1 shows the polarization and X-ray diffraction (XRD) image of P(VDF-TrFE), which illustrate the formation of β-phase P(VDF-TrFE). The ferroelectric materials' coercive voltage ($V_c$) can be served as the threshold voltage for the non-volatile memory and optoelectronics. And the diffraction peak of 19.9° shows (200) and (110) crystalline planes in the ferroelectric β phase crystallinity.

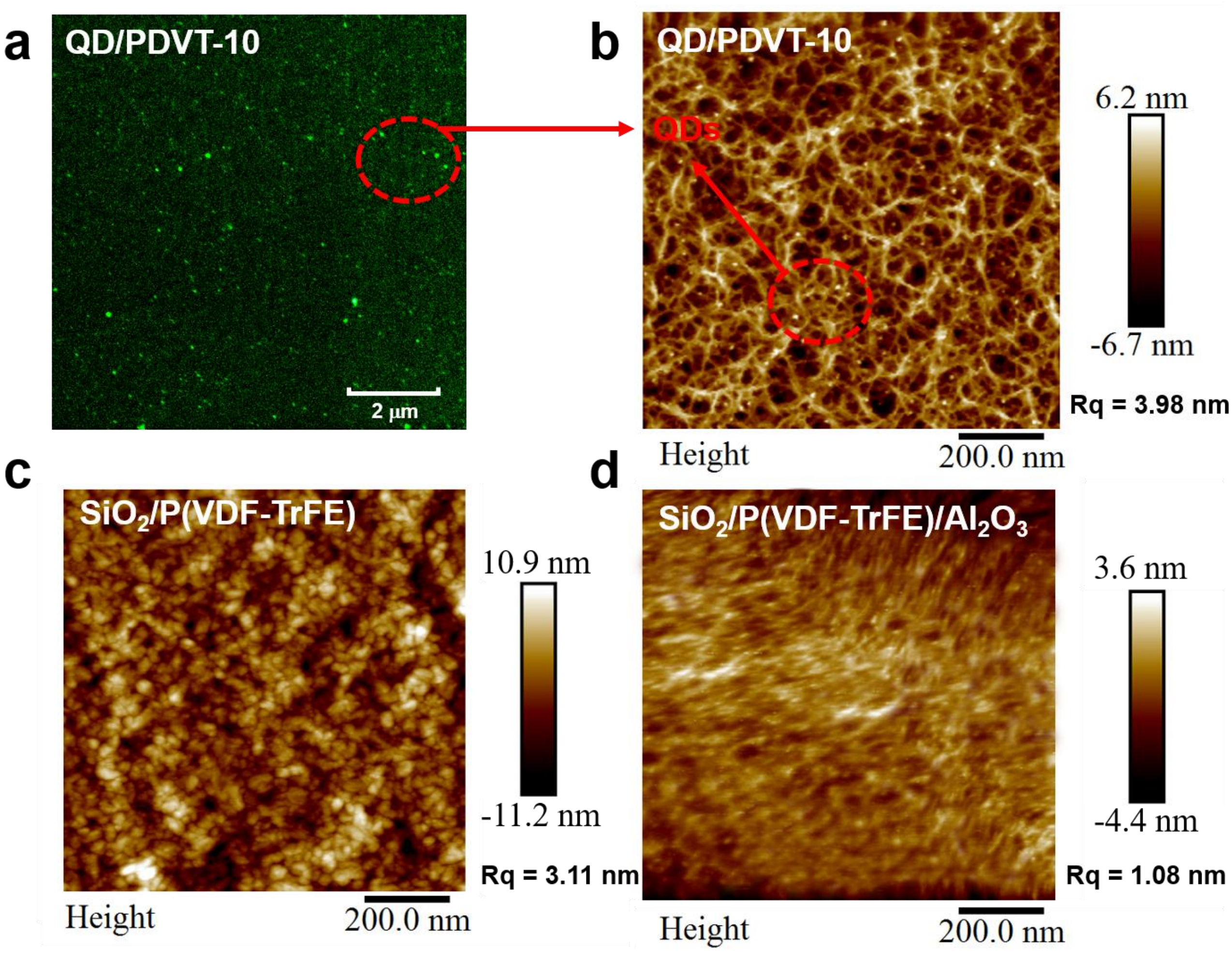


**Figure S2 The morphological characterizations of films.** a) The fluorescence images of QDs distributed in the composited film. The atomic forcemicroscopy (AFM) images of b) QDs/PDVT-10 composited film c) the $SiO_2$/P(VDF-TrFE) structure d) the $SiO_2$/P(VDF-TrFE)/$Al_2O_3$ structure.

The composited film is observed under a fluorescence microscope and AFM (Fig. S2 a and b), the QDs can be clearly observed and the QDs are evenly distributed. As shown in Fig. S2c and d, the $SiO_2$/P(VDF-TrFE) and $SiO_2$/P(VDF-TrFE)/$Al_2O_3$ morphological characterizations (AFM) have shown that the morphology with $Al_2O_3$ layer (RMS = 1.08 nm) is much better than that without $Al_2O_3$ layer (RMS = 3.11 nm).

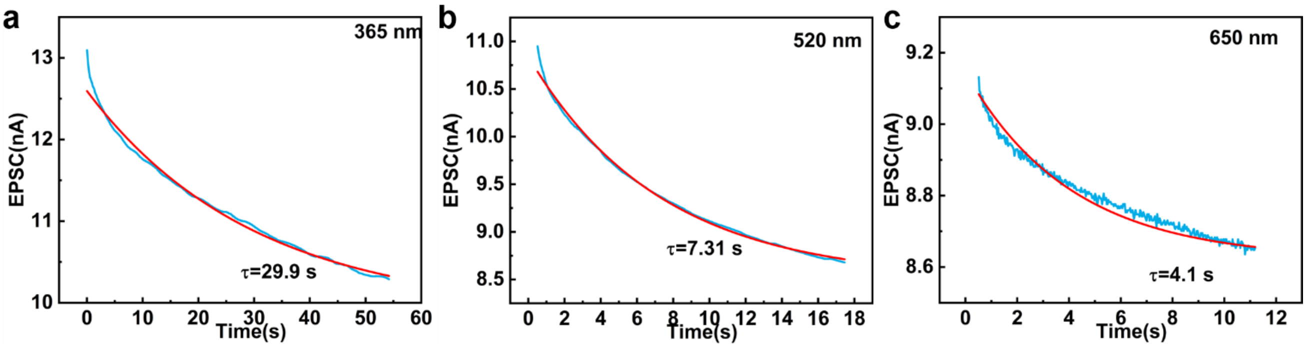


**Figure S3** The fitting curves of EPSC triggered by light spikes with wavelengths of 365 nm, 520 nm and 650 nm.

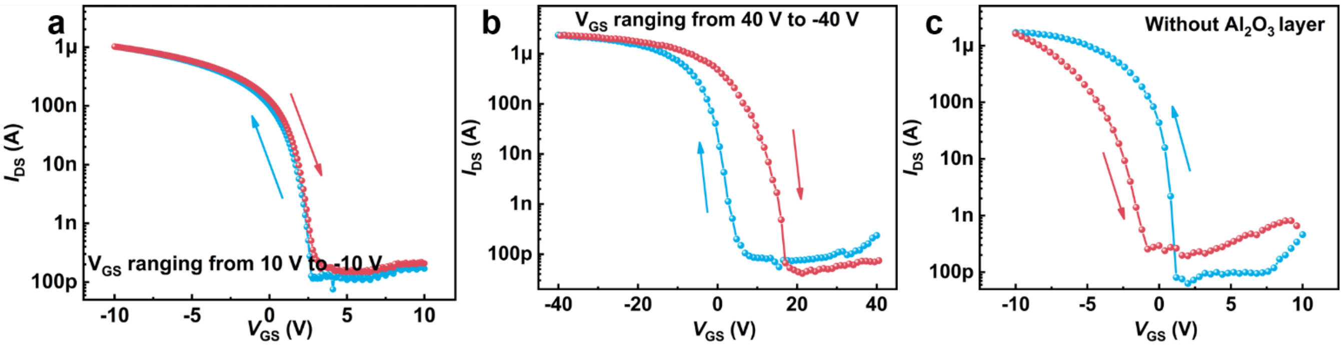


**Figure S4** The double sweeping curves with gate voltage ranging from a) −10 V to 10 V. b) -40 V to 40 V. c) The double sweeping curves the device without $Al_2O_3$ layer.

As shown in Fig. 4a, the double sweeping curves shows little clockwise hysteresis due to the partial polarization of ferroelectric layer, while a larger gate voltage range is swept in Fig. 4b, an obvious hysteresis obtained by polarization can be observed, which can be explained by coercive voltage ($V_c$). [1,2] When the volatage is lower than $V_c$, the ferroelectric materials will not generate polarization in the same direction, while gate voltage exceeds coercive voltage (Vc) in the ferroelectric material, the ferroelectric material occurs polarization and the hysteresis is generated. As shown in Fig. S3c, the double sweeping curves of ferroelectric device without $Al_2O_3$ layer show a counterclockwise hysteresis loop, indicating the trapping effect between the composite and ferroelectric film. The insertion of $Al_2O_3$ layer can effectively remove the influence of trapping effect on channel conductance. [3]

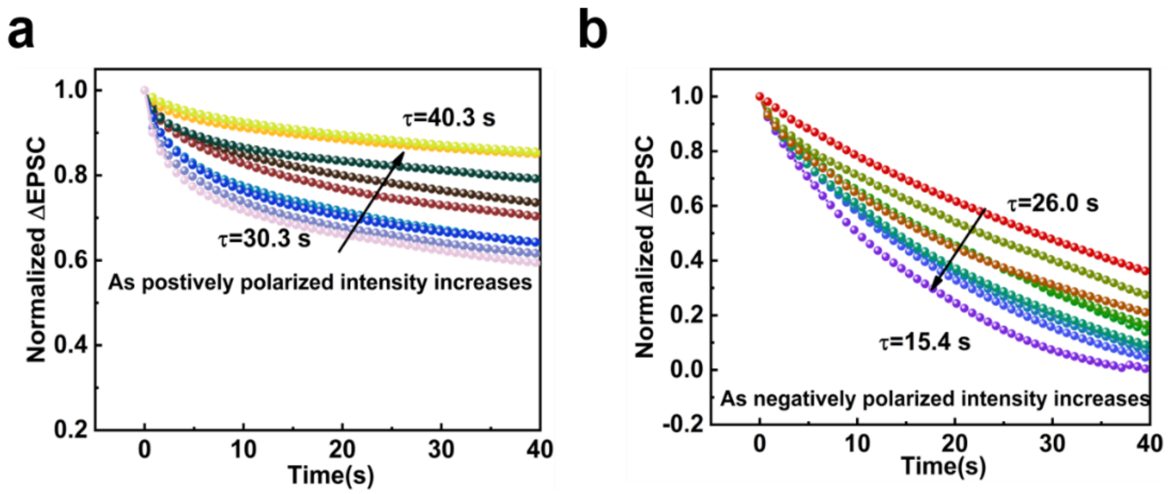


**Figure S5** Normalized ΔEPSCs in a) positive and b) negative polarization.

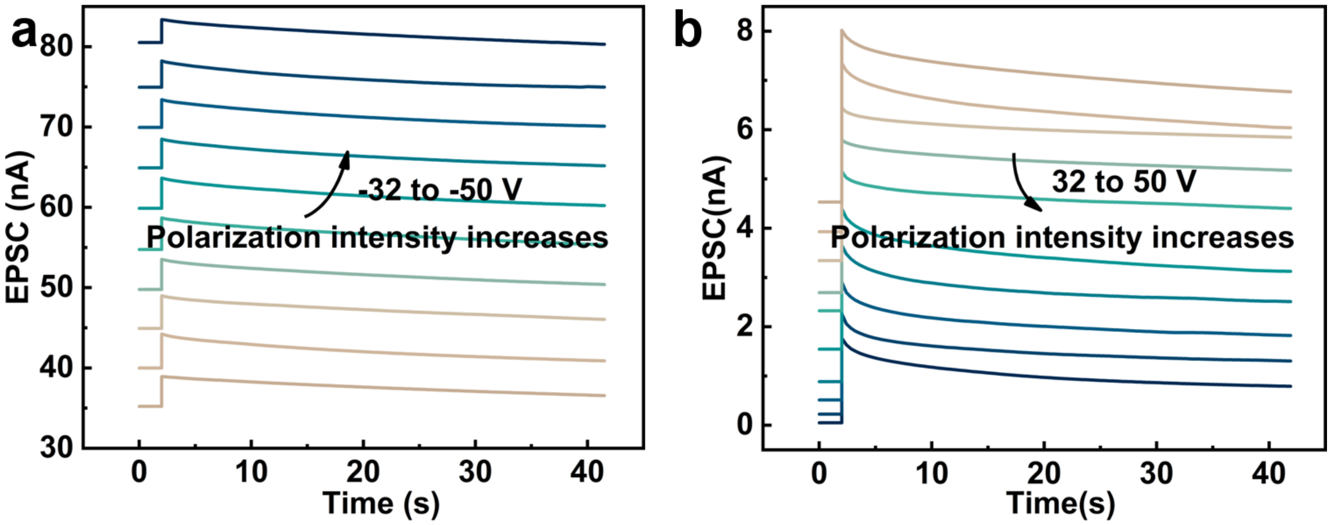


**Figure S6** The excited postsynaptic current triggered by 100 ms light pulse in a) positive polarization (32 to 50 V) and b) negative polarization (–32 to –50 V).

The 10 states of EPSCs in positive polarization are measured by applying 10 different positive bias pulses (32 to 50 V) to gate electrode. As the amplitude of positive bias increases, the polarization state of ferroelectric layer changes gradually from initial state to positive state, which results in decrease of current while the increase of the retention time. In contrast, the 10 states of EPSCs in negative polarization is presented in Fig. S6b. The current increases and the decay constant decreases with the increase of polarization intensity.

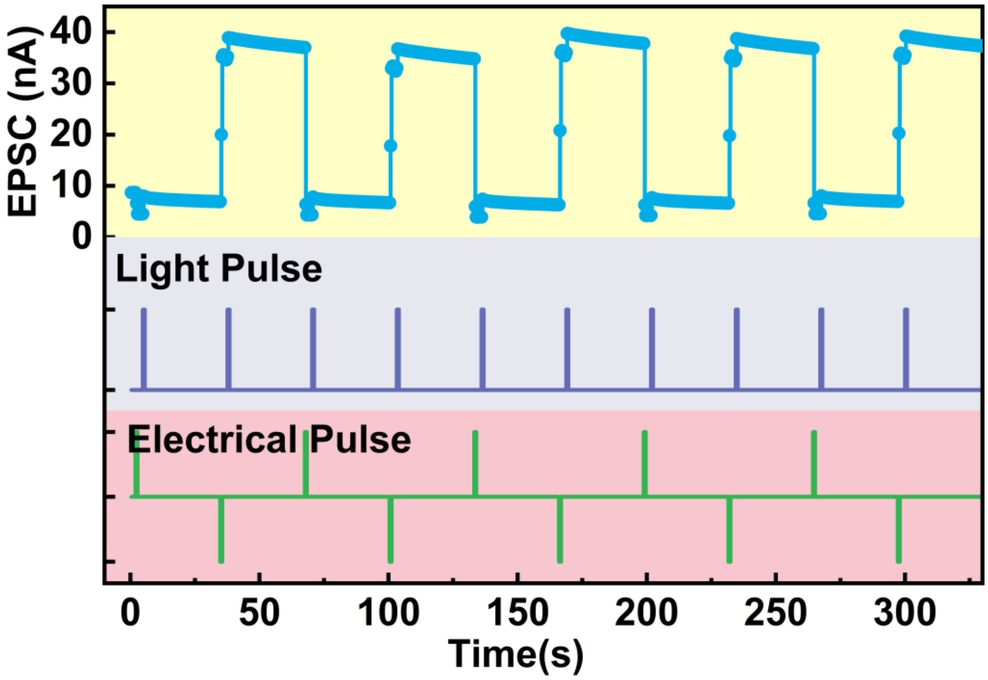


**Figure S7** EPSC after light pulse under the alternative polarization.

It illustrates the positive-negative polarization switching cycles for photoelectric memory functions. The positive and negative polarization are performed alternatively. The retention current is recorded in Fig. 3b.

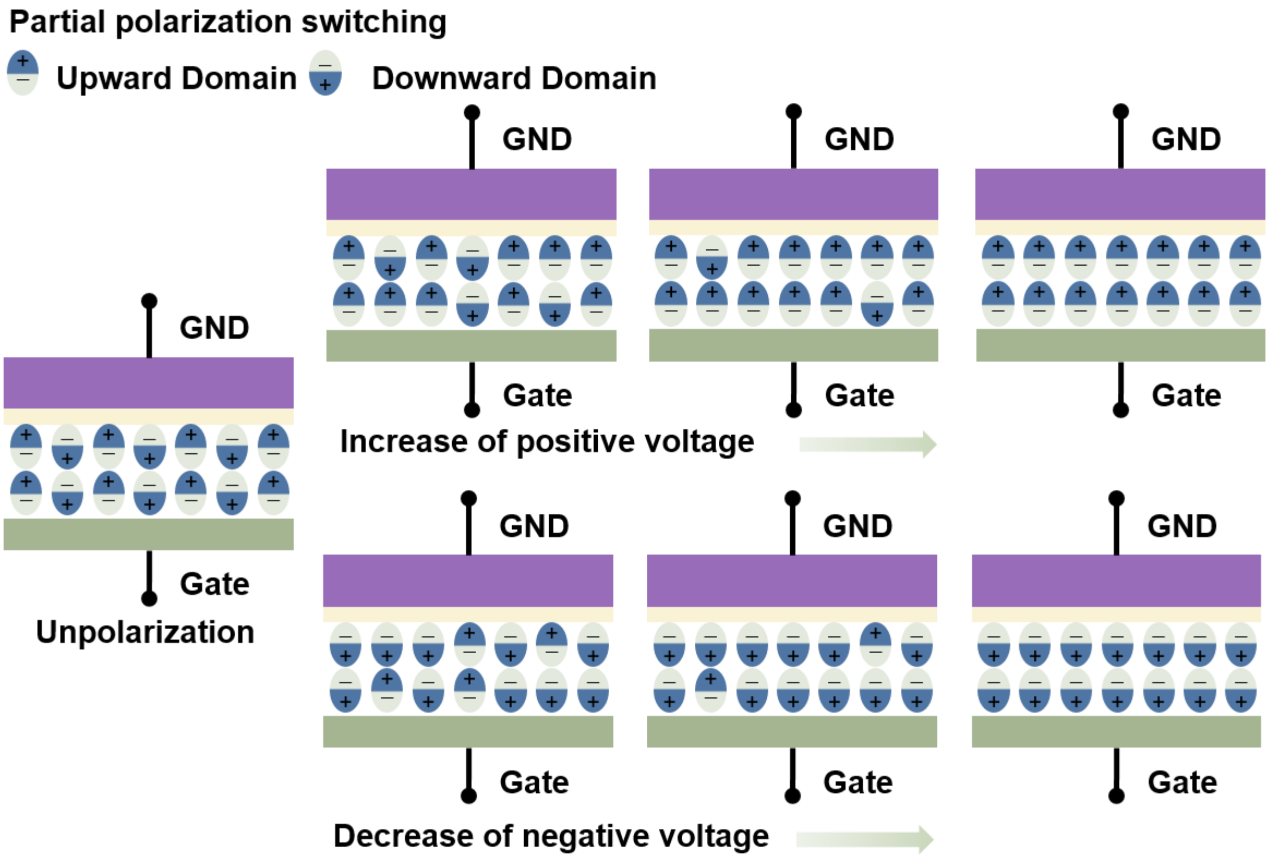


**Figure S8** Graphical illustration of partial polarization switching by positive and negative voltages

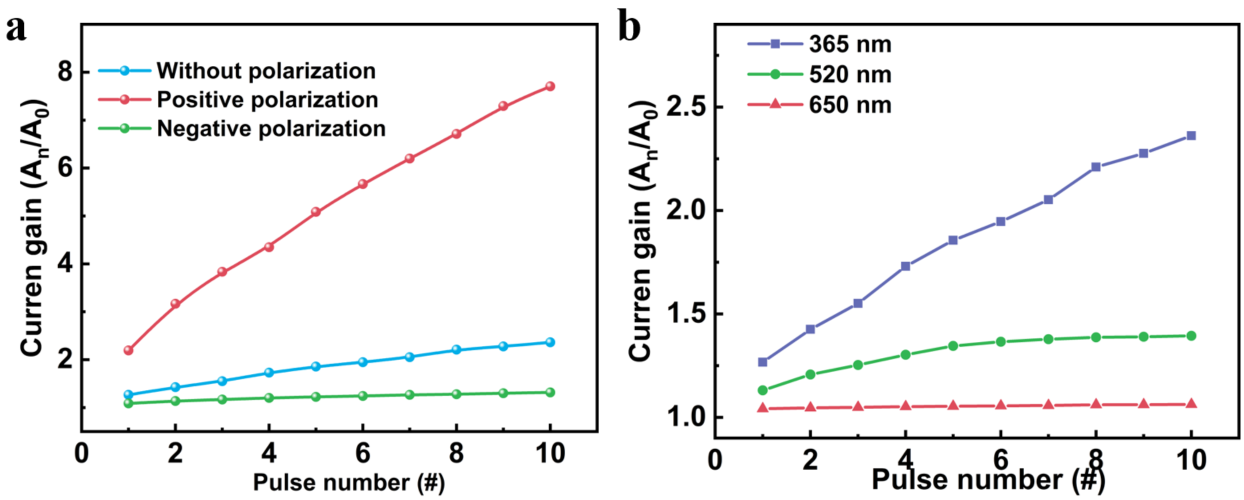


**Figure S9** a) The current gain to 10 consecutive light pulses (1 μWcm$^{-2}$, 100 ms) with and without the polarization. b) The current gain to 10 consecutive light spikes (1 μWcm-2, 100 ms) with wavelengths of 365 nm, 520 nm and 650 nm.

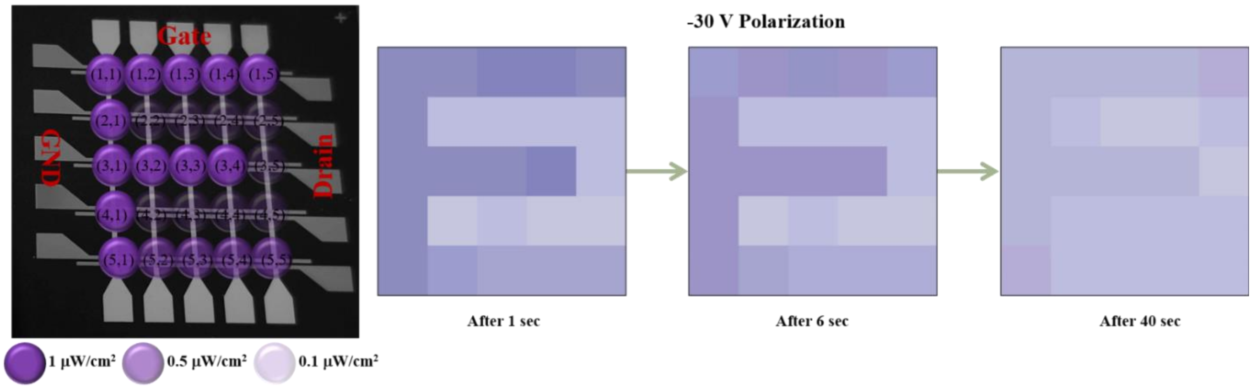


**Figure S10** Optical microscopic image of the 5×5 perception array and the encoded images (output current) after polarization.

The encoded images aresummarized from each pixel in the 5×5 sensory circuit arrays as illustrated in Figure S10 (negative polarization) and S11 (positive polarization), and the position is presented as above. The encoded images record the current after 1 s, 6 s and 40 s. To quantify the ability to identify signals, the signal to noise ratio (SNR) is presented, which defined as

$$\mathrm{SNR} = 20\lg(\frac{S}{N}) \tag{S1}$$

where *S* and *N* are the currents of signal and noise. Here, the currents of signal and noise are average current in pixel of valid signal and noise. *S* and *N* can be calculated as below

$$S = \frac{1}{N_\mathrm{S}}\sum_{i}^{P_\mathrm{S}} I_i \tag{S2}$$

$$N = \frac{1}{N_\mathrm{N}}\sum_{i}^{P_\mathrm{N}} I_i \tag{S3}$$

Here $N_\mathrm{S}$ and $N_\mathrm{N}$ are the pixel number of signal and noise. $P_\mathrm{S}$ and $P_\mathrm{N}$ are the position set of signal and noise. $I_i$ is the current in every pixel. When perception array is exposed to "E" signal, the signals are all pixels exposed by both weak and strong light ($N_\mathrm{S}$=16, $N_\mathrm{N}$=9 and PS={(1,1), …(1,5), (2,1), (3,1), …(3,4), (4,1), (5,1), …(5,5)}, PN={(2,2), …(2,5), (2,1), (3,5), (4,1), …(4,5)}). The calculation results are shown in Figure 5e and f.

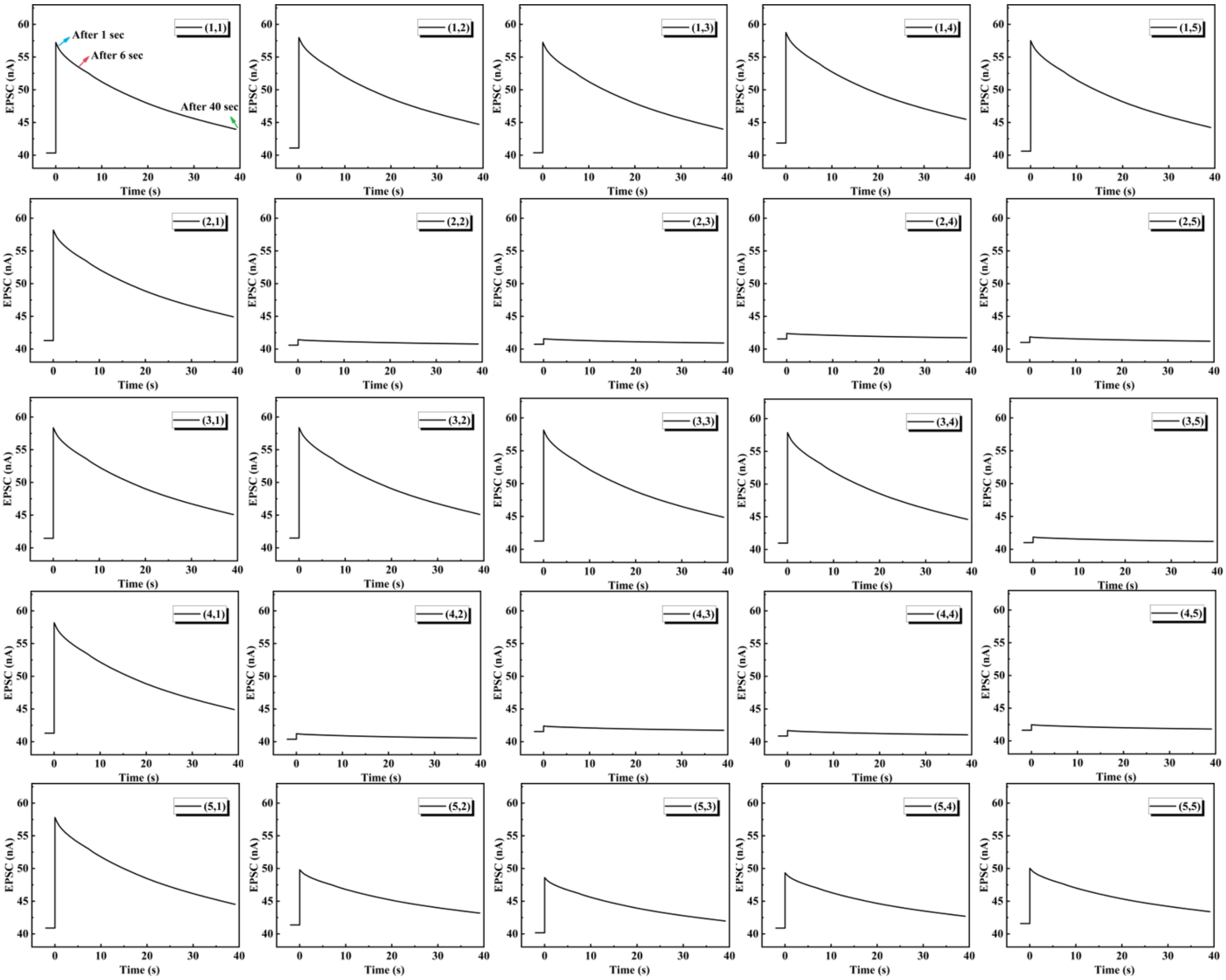


**Figure S11** EPSC of each pixel in the 5×5 perception array after -30V polarization.

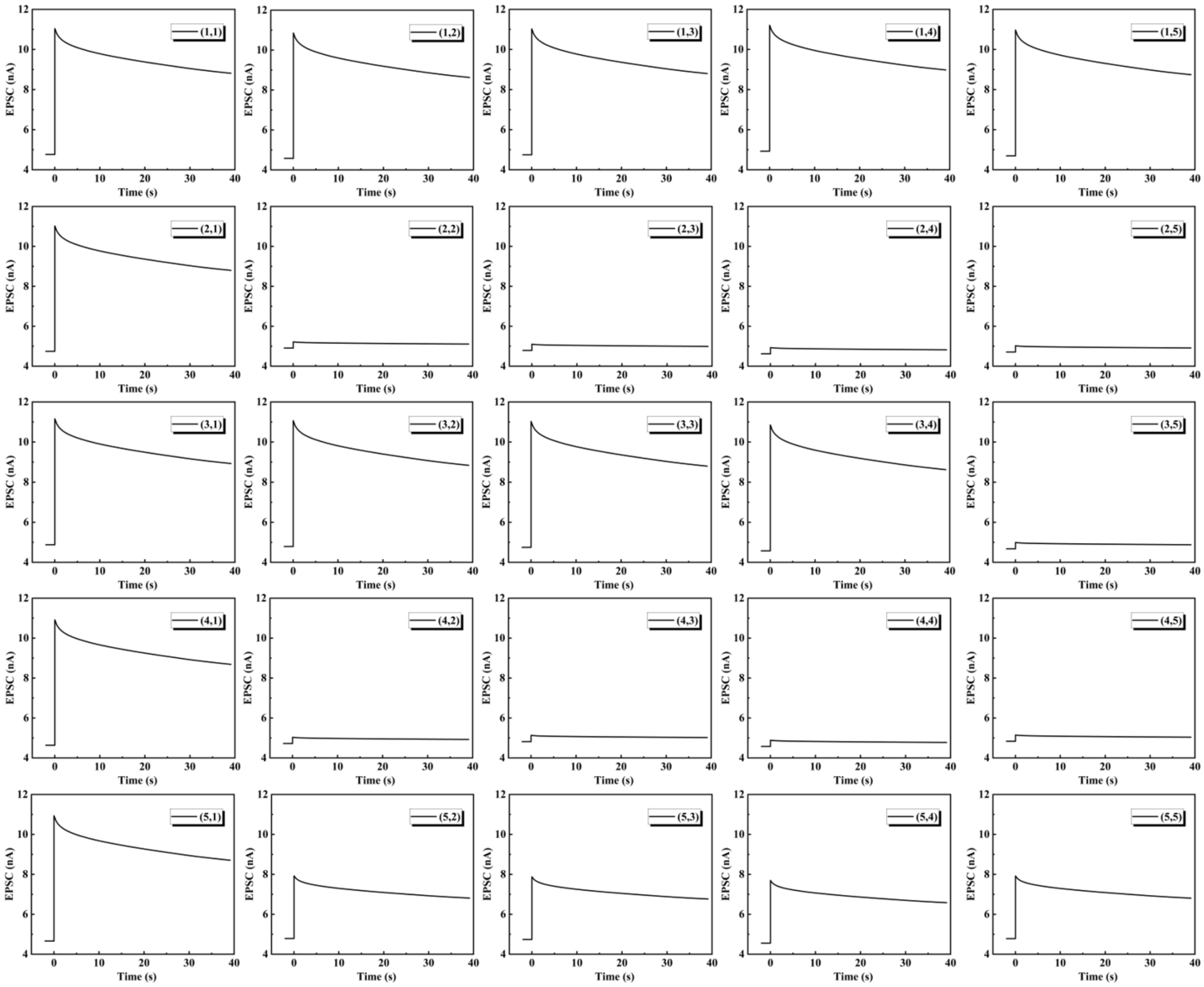


**Figure S12** EPSC of each pixel in the 5×5 perception array after 30V polarization.

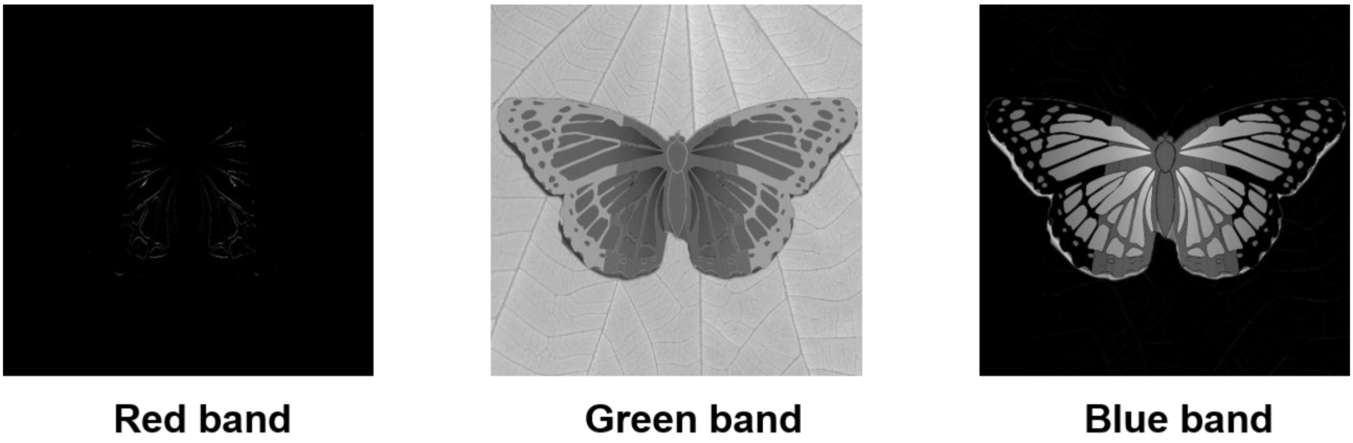


**Figure S13** Demonstration of different image in different monochrome bands (650, 520 and 420 nm).

An image can be divided into three gray images with three monochrome bands of 650, 520 and 420 nm, similar to images achieved by our devices that work in a single wavelength band. Due to the wavelength dependence, the conductance can be obtained by sum of conductance of the device produced by the three monochrome light, which is simulated by the MATLAB software.

**Hardware implementations of neural network.**

The most frequent large hardware implementations of deep neural networks are built by analogue vector-matrix multiplier (VMM) circuits. The considered circuits are shown in Fig. S14, where each ferroelectric transistor is used as sensor and programmable resistor. During inference, the control drain voltages are set at $V_{mn}$ ($m$ =1,2,3; $n$ =1,2,3), which represent weights ($w_{mn}$) in neural network. And the input conductance modulated by light pulses can be expressed as $G_{mn}$($m$ =1,2,3; $n$=1,2,3), which input vectors ($x_{mn}$) in neural network. Then the output current ($I$) is given by matrix multiplication of the voltage, which represents the output ($y$) in the neural network. The out of neural network can be described as

$$y = \sum_{m=1,n=1}^{3} w_{mn}x_{mn} + b \tag{S4}$$

Since the drain current is linear in the measured range (Fig. S15), according to Kirchhoff's and Ohm's laws, the output current can be described as

$$I_{out} = \sum_{m=1,n=1}^{3} V_{mn}G_{mn} + V_0/R \tag{S5}$$

which is similar to the equation S4. Hence, the neural network is constructed by simple VMM circuits.

In the training epoch, the input pattern is encoded to conductance and incorporated into the neural network to update the drain voltage. The flow charts of training and testing process are demonstrated in Fig. S16. The training process aims to train appropriate weight values to decrease the loss function (Fig. S17).

Compared to the recognition results of the network with selective attention in the main manuscript, the results without selective attention exhibit low recognition accuracy. The encoding conductance of pattern with variant wavelengths and the distribution of the output accumulated current in the 5 input patterns are supplied in the Fig. S18a. Its exhibits low contrast in the conductance. The distribution of the output currents are indistinguishable, so the accuracy without selective attention is far lower than the accuracy with selective attention (Fig. S18b).

The neural network proposed in the recent are mostly based on sensory and computing array. [4-11] The sensor module is separated from computing module. As shown in Fig. S19, the proposed neural network need $m$ sensors and $m \times n$ synapses to realize recognition task including $m$ input pixels and n target patterns.

But in our proposed work, the sensor and computing function are integrated in the same region, only *m* ferroelectric transistors are required to construct the network. So it illustrates the superiorities of its work that is less hardware overhead.

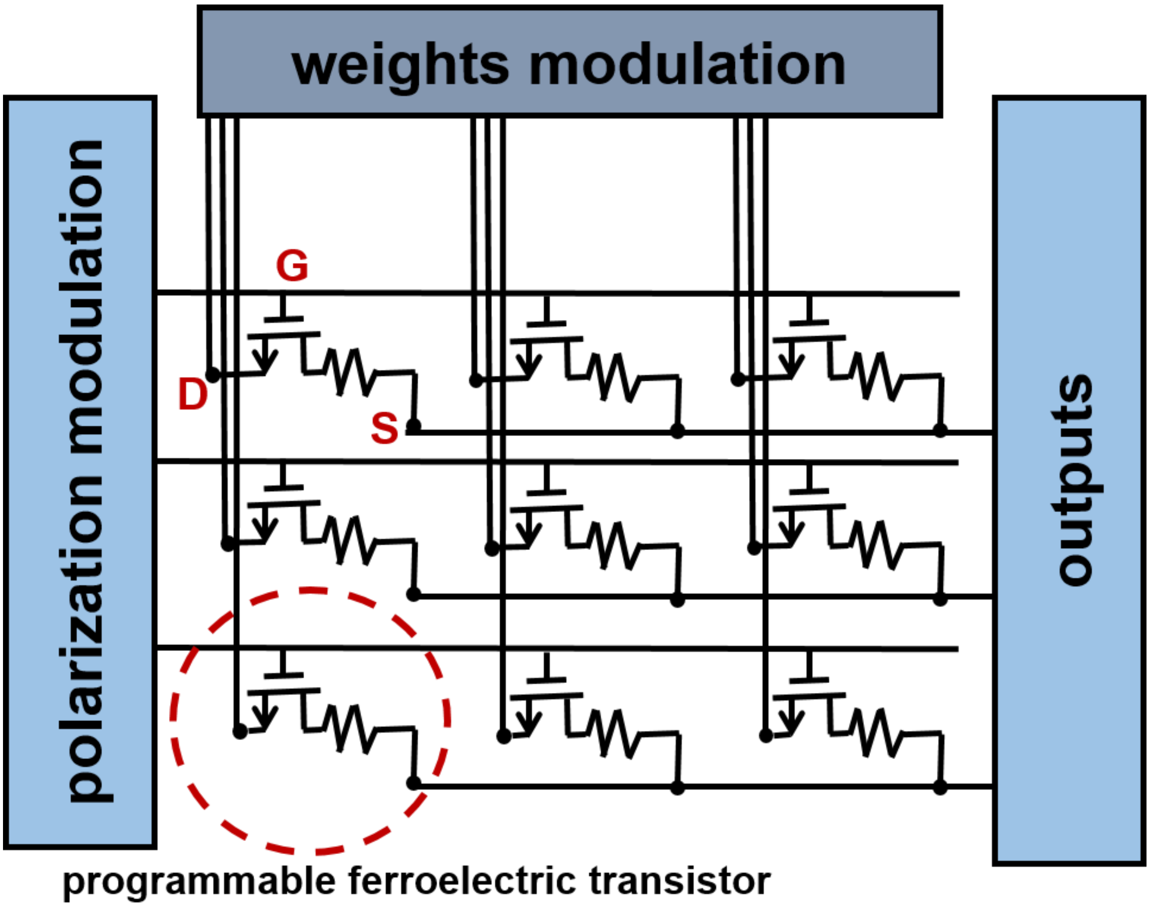


**Figure S14** Vector-matrix multiplier (VMM) circuits in the hardware implementations of deep neural networks.

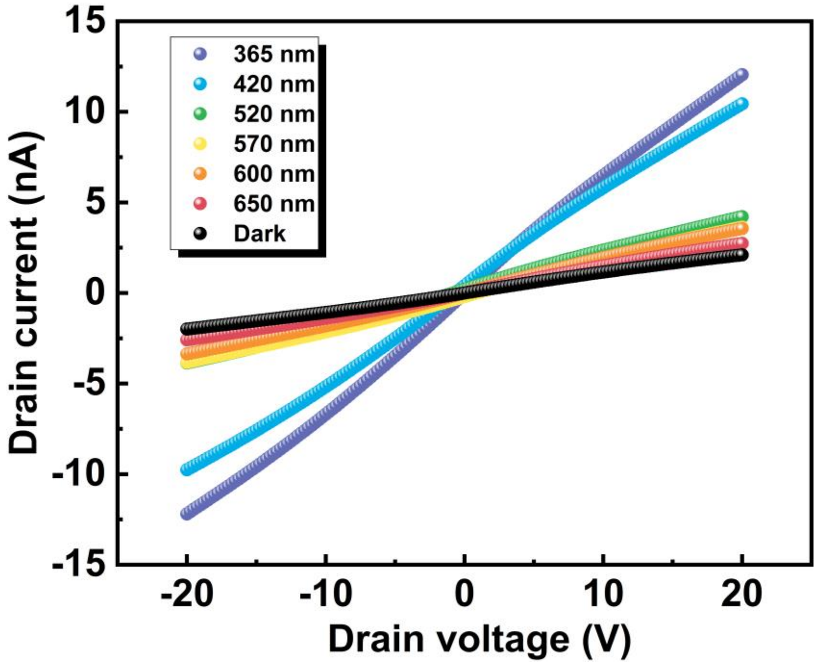


**Figure S15** The output curves in dark and UV illumination condition.

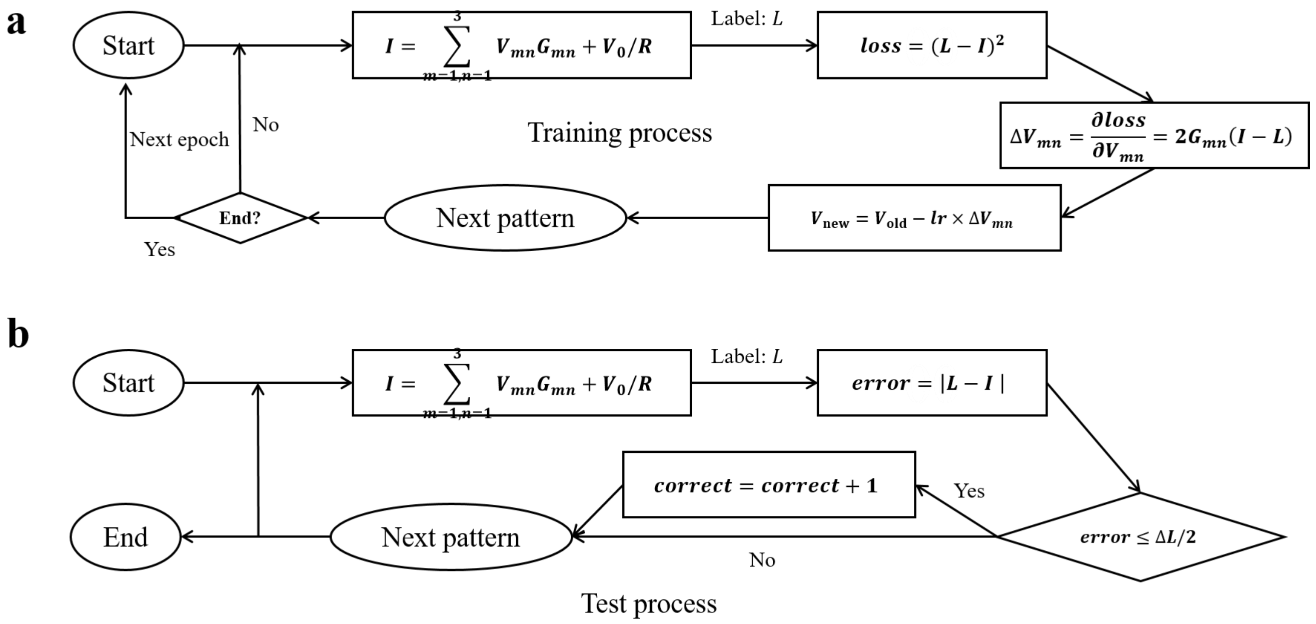


**Figure S16** The flow chart in the training and testing process.

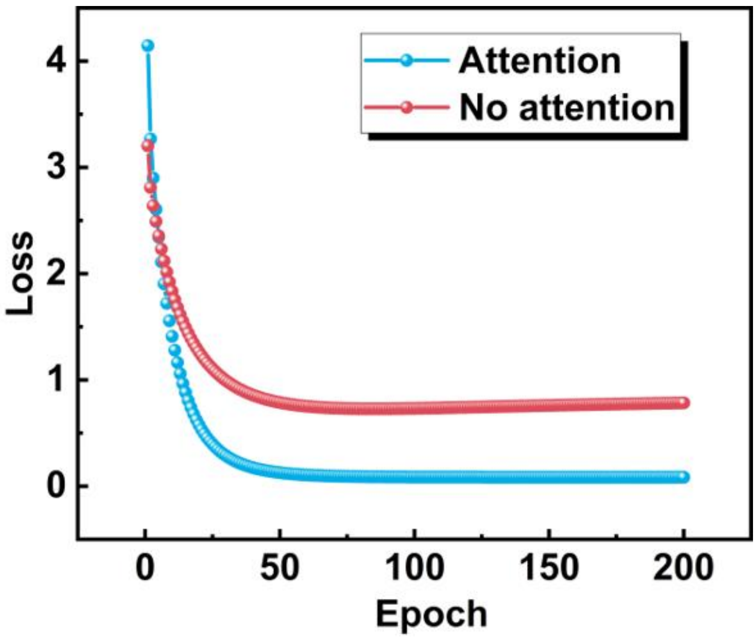


**Figure S17** The loss function as epochs increases.

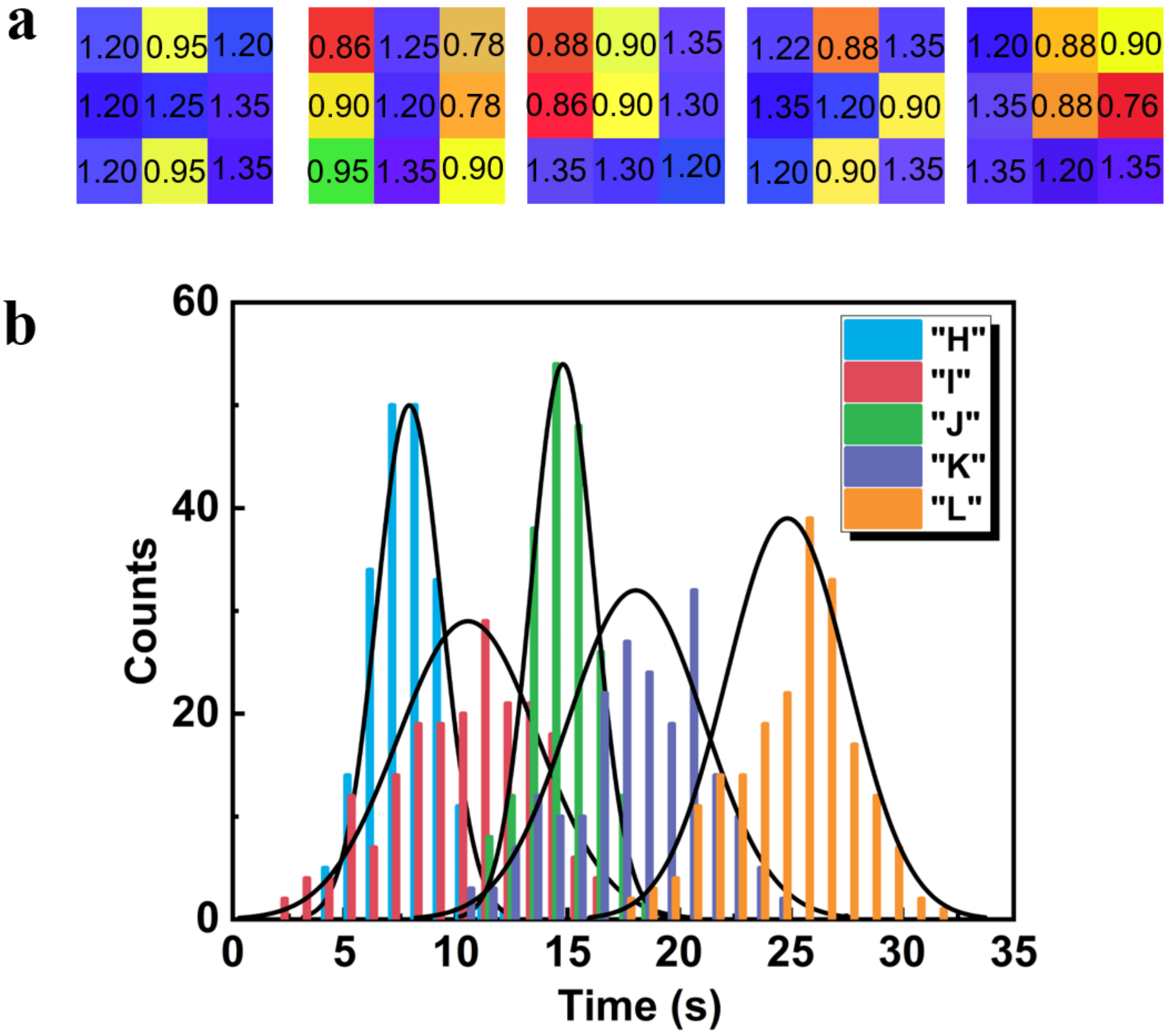


**Figure S18** a) The encoding conductance of pattern with variant wavelengths and b) the distribution of the output accumulated current in the 5 input patterns, which is test in the device without attention.

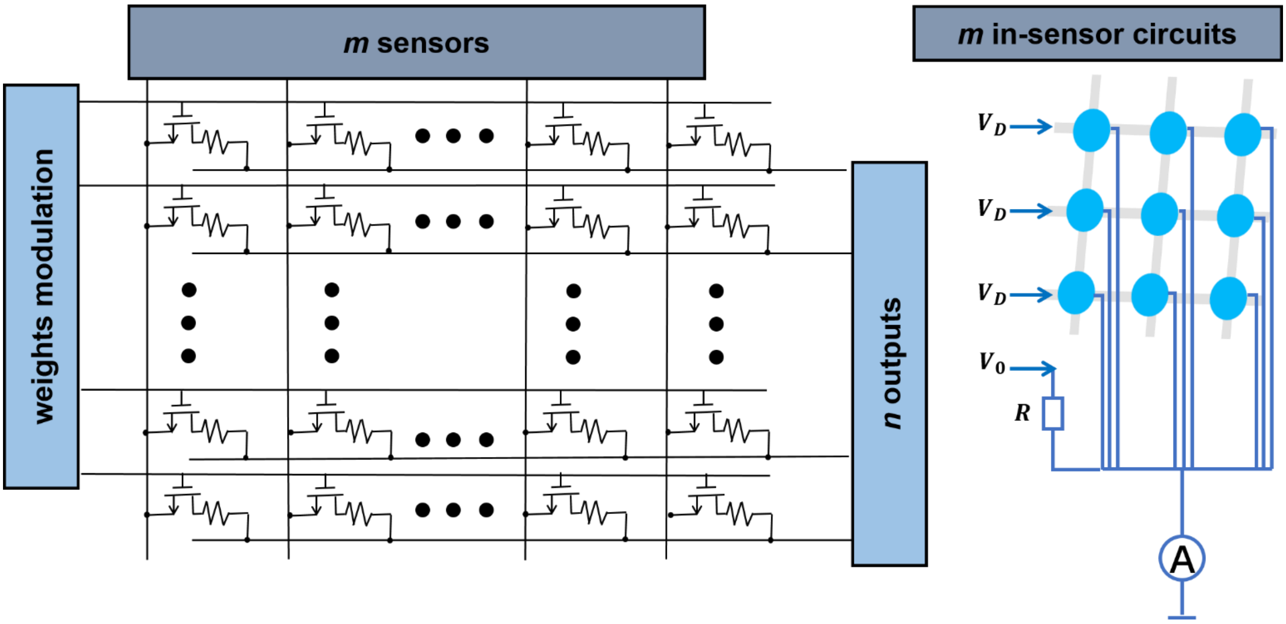


**Figure S19** The comparison between recent neural network and proposed neural network in this work.

| Drain voltage ($V_{mn}$) | 1 | 2 | 3 |
|---|---|---|---|
| 1 | 0.60 V | -2 V | 5.67 V |
| 2 | 0.08 V | -6.80 V | -24.69 V |
| 3 | 9.35 V | 9.04 V | 9.17 V |
| Bias current ($V_0/R$) | 10.7 nA | | |

**Table S1.** The set drain voltage and the bias current after training.

## Supplementary References


1. Wang, H. et al. A ferroelectric/electrochemical modulated organic synapse for ultraflexible, artificial visual-perception system. *Adv. Mater.* **30**, 1803961 (2018).
2. Kim, M. K. & Lee J. S. Ferroelectric analog synaptic transistors. *Nano Lett.* **19**, 2044-2050 (2019).
3. Shi, D.-k. et al. Improving the barrier inhomogeneity of 4h-sic schottky diodes by inserting al2o3 interface layer. *Solid-State Electronics* **180**, 107992 (2021).
4. Li, S. et al. Wafer-scale 2D hafnium diselenide based memristor crossbar array for energy-efficient neural network hardware. *Adv. Mater.*, e2103376 (2021).
5. Tang, J. et al. Bridging biological and artificial neural networks with emerging neuromorphic devices: Fundamentals, progress, and challenges. *Adv. Mater.* **31**, e1902761 (2019).
6. Choi, S. et al. Sige epitaxial memory for neuromorphic computing with reproducible high performance based on engineered dislocations. *Nat. Mater.* **17**, 335-340 (2018).
7. van de Burgt, Y. et al. A non-volatile organic electrochemical device as a low-voltage artificial synapse for neuromorphic computing. *Nat. Mater.* **16**, 414-418 (2017).
8. Kim, S. et al. Pattern recognition using carbon nanotube synaptic transistors with an adjustable weight update protocol. *ACS Nano* **11**, 2814-2822 (2017).
9. Kim, S., Yoon J., Kim H. D. & Choi S. J. Carbon nanotube synaptic transistor network for pattern recognition. *ACS Appl Mater Interfaces* **7**, 25479-25486 (2015).
10. Liu, J. et al. Compensated ferrimagnet based artificial synapse and neuron for ultrafast neuromorphic computing. *Adv. Func. Mater.*, 2107870 (2021).
11. Yang, C.-S. et al. All-solid-state synaptic transistor with ultralow conductance for neuromorphic computing. *Adv. Func. Mater.* **28**, 1804170 (2018).
